# APC regulates NSLIT2-induced internalization and signaling of the chemorepellent receptor, ROBO1

**DOI:** 10.26508/lsa.202403190

**Published:** 2026-11-10

**Authors:** Yi-Wei Huang, Zhubing Li, Jonathan St-Germain, Bo Wen Pang, Richard F Collins, Etienne Coyaud, Wenjuan Li, Sajedabanu Patel, Nourhan Almasri, Amir Mohamed, Brian Raught, Sergio Grinstein, Lisa A Robinson

**Affiliations:** 1 Program in Cell and Systems Biology, Hospital for Sick Children Research Institute , Toronto, Canada; 2 Princess Margaret Cancer Centre, University Health Network, Toronto, Canada; 3 Department of Medical Biophysics, University of Toronto , Toronto, Canada; 4 Institute of Medical Science, University of Toronto , Toronto, Canada; 5 Department of Biochemistry, University of Toronto , Toronto, Canada; 6 Division of Nephrology, The Hospital for Sick Children , Toronto, Canada; 7 Department of Paediatrics, University of Toronto , Toronto, Canada; 8 PRISM INSERM U1192, Université de Lille , Villeneuve d’Ascq, France

## Abstract

The SLIT–Roundabout signaling pathway regulates axon guidance and cell migration, and ROBO1 is a receptor for SLIT ligands. ROBO1 undergoes constitutive endocytosis, which is enhanced upon NSLIT2 binding, but the molecular mechanisms and functional consequences of this process are not well understood. Using pharmacologic inhibitors and molecular techniques, we found that clathrin-mediated endocytosis is necessary for NSLIT2-induced inhibition of cell spreading. To explore the underlying mechanisms, we performed proximity-dependent biotin identification to identify ROBO1-interacting proteins whose association with the cytoplasmic domain of ROBO1 is differentially regulated by NSLIT2. We discovered that adenomatous polyposis coli, a multifunctional tumor suppressor, is in close proximity to ROBO1 but dissociates upon binding of NSLIT2 and that this dissociation is necessary for clathrin-mediated endocytosis of ROBO1 and subsequent effects on cell morphology. These findings provide new insights into the functional mechanisms by which NSLIT2 binding to ROBO1 affects changes in actin cytoskeletal architecture.

## Abstract (precis)

SLIT2 stimulation triggers ROBO1 internalization by disengaging the tumor suppressor APC, enabling clathrin-mediated endocytosis and cytoskeletal remodeling that regulates cell shape and movement.

## Introduction

ROBO receptors are a family of highly conserved axon guidance receptors, which together with their specific secreted SLIT ligands play a repulsive role to prevent axons from crossing the midline repeatedly and randomly during neuronal development (Brose et al, 1999; Kidd et al, 1999). SLIT-ROBO signaling also negatively regulates adhesion, spreading, and migration of diverse hematopoietic cell types, including neutrophils, platelets, and macrophages (Tole et al, 2009; Patel et al, 2012), and inhibits macropinocytosis in macrophages (Shi et al, 2004; Zhou et al, 2004; Bhosle et al, 2020). SLIT-ROBO regulate actin cytoskeletal remodeling through the inhibition of Rac1 and Cdc42 (Tole et al, 2009) or the activation of RhoA (Bhosle et al, 2020) via the binding of SLIT-ROBO GTPase-activating proteins or inactivation of the Rho GTPase–activating protein, myosin IXb, respectively (Wong et al, 2001; Guerrier et al, 2009).

In the developing nervous system of *Drosophila*, repulsion of midline axons has been demonstrated to require SLIT-induced clathrin-mediated endocytosis (CME) of ROBO from the cell surface (Chance & Bashaw, 2015; Howard et al, 2019). In murine cells, endocytosis and recycling of ROBO1 are required for SLIT-induced responses and further sensitization in vertebrate commissural axons upon midline crossing (Kinoshita-Kawada et al, 2019). However, the underlying molecular mechanisms of CME of ROBO in mammalian cells are relatively unexplored. We report here that in mammalian cells, binding of the bioactive N-terminal region of SLIT2, NSLIT2, to ROBO1 induces endocytosis of ROBO1 in a dynamin- and clathrin-dependent manner and that endocytosis of ROBO1 from the cell surface requires the dissociation of the tumor suppressor protein, APC. We found that APC is in close proximity to the intracellular domain of ROBO1 and that binding of NSLIT2 to ROBO1 causes APC to dissociate from ROBO1. The functional consequences of APC disengagement from ROBO1 on NSLIT2-induced cytoskeletal changes are further discussed.

## Results

### ROBO1 undergoes basal endocytosis, which is enhanced by the binding of the ligand, NSLIT2

**Figure S1.**
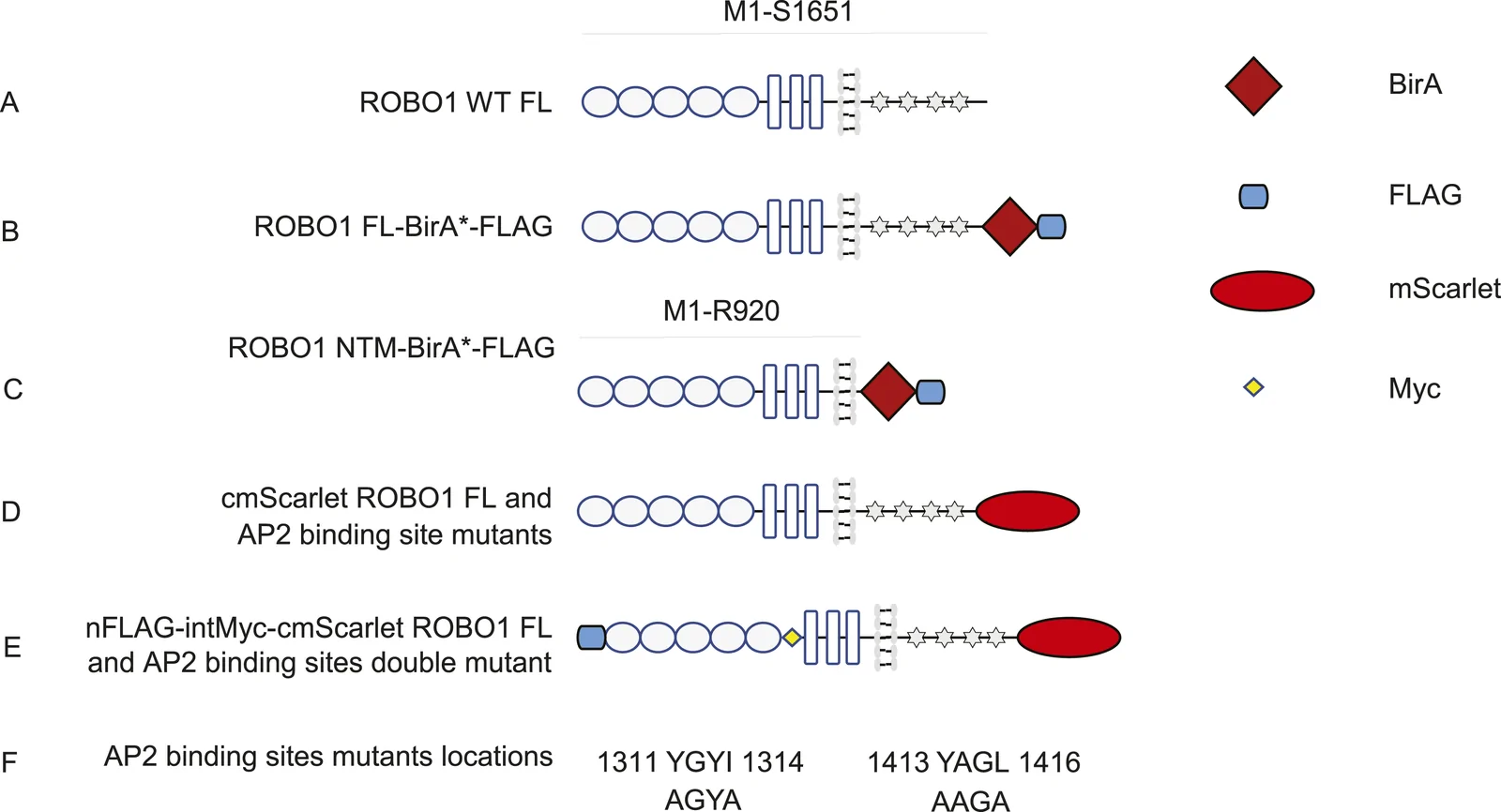
Schematic of ROBO1 cDNA plasmids. **(A)** represents the original YFP-tagged ROBO1 FL plasmid (pYFP C1-ROBO1 FL) and an intermediate state of pzeo-/ROBO1 FL used to generate plasmids for BioID experiments. **(B)** represents the pcDNA5 FRT/TO cBirA-FLAG ROBO1 FL plasmid. **(C)** represents the pcDNA5 FRT/TO cBirA-FLAG ROBO1 NTM (N-terminal mutant) plasmid. **(D)** represents pcDNA zeo+/cmScarlet-ROBO1 FL, pcDNA zeo+/cmScarlet-ROBO1-AGYA or pcDNA zeo+/cmScarlet-ROBO1-AAGA, or pcDNA zeo+/cmScarlet-ROBO1-AP2-binding site double mutant plasmids. **(E)** represents pcDNA zeo+/n FLAG-intMyc-cmScarlet-ROBO1 FL or pcDNA zeo+/n FLAG-intMyc-cmScarlet-ROBO1-AP2-binding site double mutant plasmids. **(F)** Location of AP2-binding motifs in ROBO1.

**Figure 1.**
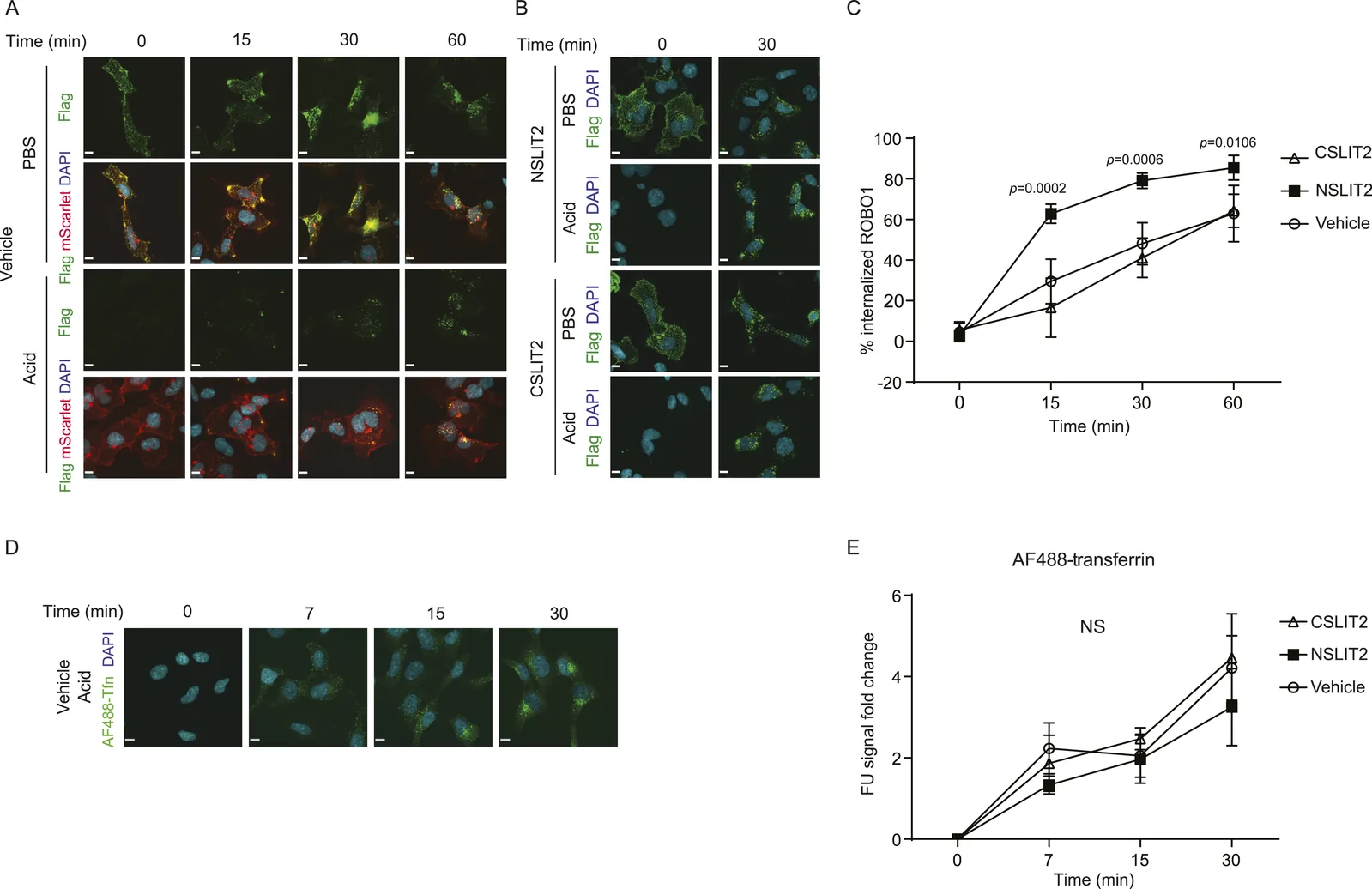
ROBO1 undergoes constitutive endocytosis, which is augmented by exposure to NSLIT2. **(A)** 5H9 cells on coverslips were treated with the ADAM10 inhibitor GI 254023X (10 μM) overnight to maximize cell surface levels of ROBO1, then incubated with anti-FLAG antibody for 1 h on ice. Cells were warmed to 37°C to allow endocytosis. At indicated time points, cells were subjected to acid wash or wash with PBS, fixed and permeabilized, then labeled with AF488-conjugated goat anti-mouse secondary antibody. Images were acquired using Quorum Spinning Disk Confocal microscope Leica DMi8. Green, N-terminal FLAG-tagged ROBO1; red, C-terminal mScarlet ROBO1. **(B)** Experiments were performed as in (A), but cells were incubated with NSLIT2 (30 nM) or equimolar concentration of CSLIT2 before initiating endocytosis at 37^ο^C. Representative images are shown at 0- and 30-min time points. **(C)** Experiments were performed as in (B), and the percentage of internalized FLAG-tagged ROBO1 was serially measured. Results are from four independent experiments. *P*-values were determined using two-way ANOVA followed by Sidak’s post hoc multiple comparison test. **(D)** Experiments were performed as in (A), but cells were incubated with AF488-conjugated Tfn to assess endocytosis of Tfn at serial time points. Representative images were shown at indicated time points. **(E)** Experiments were performed as in (D), and internalized AF488-Tfn was serially measured. Results are from three independent experiments. *P*-values were determined using two-way ANOVA followed by Sidak’s post hoc multiple comparison test. NS = not significant. Scale bars in each image are 10 μm. Tfn, transferrin.

It has been reported that SLIT-dependent endocytic trafficking of the ROBO receptor is necessary for its repulsive signaling output in *Drosophila* (Chance & Bashaw, 2015). To investigate whether human ROBO1 undergoes endocytosis, a HEK293A cell line stably expressing human ROBO1 fused with an N-terminal FLAG tag, extracellular juxtamembrane Myc tag, and C-terminal mScarlet tag (pcDNAZeo+/nFLAG-intMyc-cmScarlet-ROBO1) was established, and referred to as 5H9 cells (Fig S1E). The extracellular domain of ROBO1 undergoes constitutive shedding by the metalloprotease, A Disintegrin and Metalloprotease 10 (ADAM10) (Coleman et al, 2010). Therefore, to maximize ROBO1 levels on the surface of 5H9 cells, cells were incubated with the ADAM10 inhibitor, GI 254023X, before performing endocytosis experiments, and internalization of the FLAG signal was serially monitored (Fig 1A and B; Reiss et al, 2005). Because NSLIT2 binding to ROBO1 can facilitate ADAM10-mediated ROBO1 cleavage to release the extracellular domain of ROBO1 (Coleman et al, 2010), this may have diminished the visible signal for FLAG early on (Fig 1B). For cells exposed to vehicle or to bio-inactive CSLIT2, internalization of ROBO1 from the cell surface was ∼20% at 15 min, 40% at 30 min, and 50% at 60 min (Fig 1C). Exposure to bioactive NSLIT2 significantly increased internalization of ROBO1 at all time points, to ∼60% at 15 min, 75% at 30 min, and 80% at 60 min (Fig 1C and *P* = 0.0002, 0.0006, and 0.0106, respectively, versus vehicle or CSLIT2). To determine whether NSLIT2 enhanced the internalization of cell surface receptors other than ROBO1, we measured endocytosis of transferrin (Fig 1D). At all time points examined, internalization of transferrin was similar for cells exposed to vehicle control, CSLIT2, and NSLIT2 (Fig 1E and *P* = 0.1165, 0.9765, and 0.3331 at 7, 15, and 30 min, respectively, versus vehicle; *P* = 0.4012, 0.3585, and 0.2861, versus CSLIT2). Together, these results indicate that exposure of cells to NSLIT2 specifically enhances internalization of ROBO1 from the cell surface but does not stimulate endocytosis globally.

### NSLIT2-induced endocytosis of ROBO1 from the cell surface is clathrin-dependent

**Figure S2.**
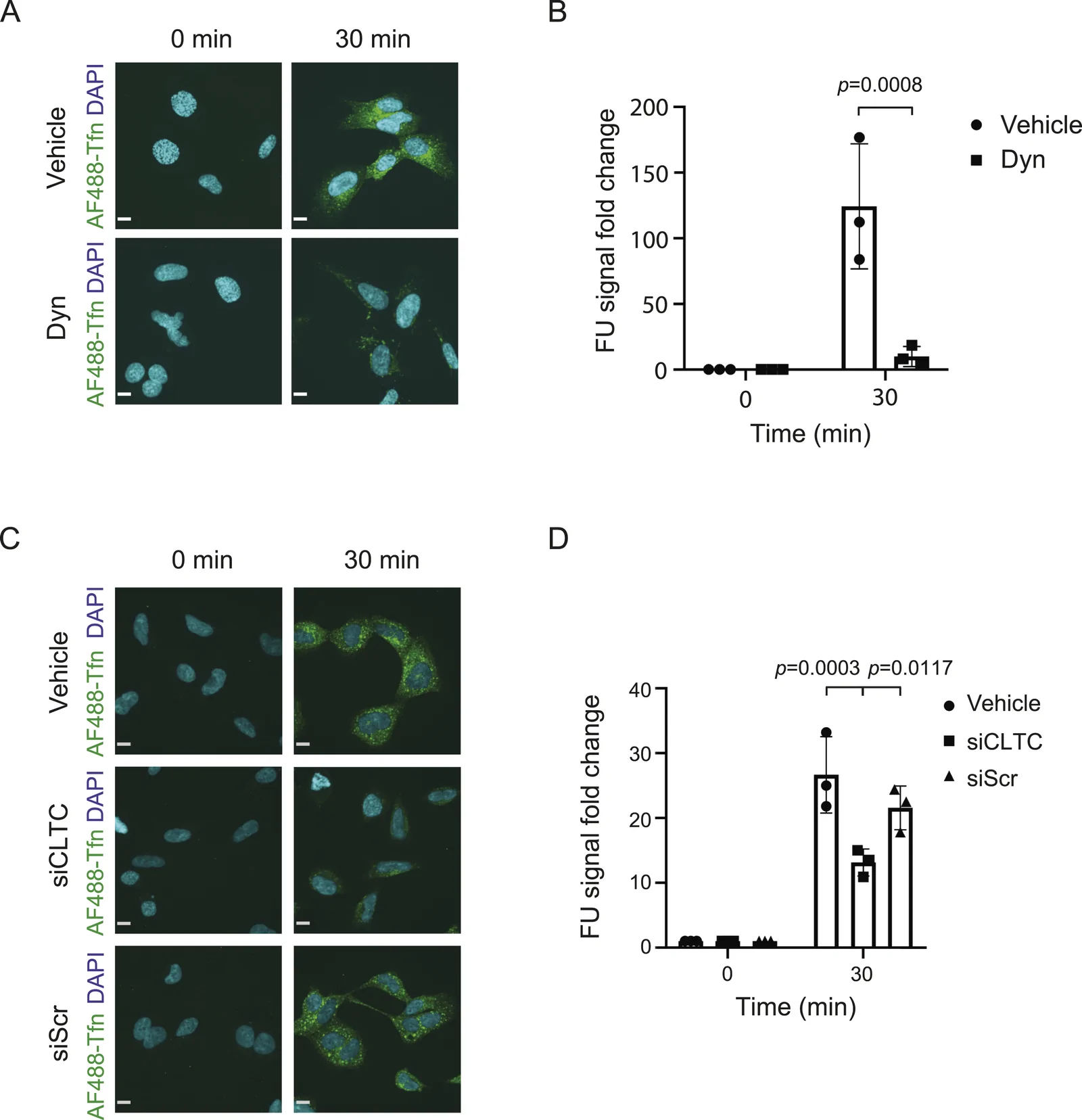
Endocytosis of Tfn is dynamin- and clathrin-dependent. **(A)** 5H9 cells were incubated with the dynamin inhibitor, Dyngo-4a (Dyn), and endocytosis of AF488-Tfn was assessed as in Fig 1D. Representative images are shown. **(B)** Experiments were performed as in (A). Results from three independent experiments were analyzed using two-way ANOVA followed by Sidak’s post hoc multiple comparison test. **(C)** Cells were incubated with specific siRNA targeting *CLTC* (siCLTC) or with scrambled, nontargeting siRNA (siScr). After 4 d, cells were incubated with AF488-conjugated Tfn to assess endocytosis of Tfn at serial time points. Representative images are shown. **(D)** Experiments were performed as in (C). Results from three independent experiments were analyzed using two-way ANOVA followed by Sidak’s post hoc multiple comparison test. Scale bars in images are 10 μm. Tfn, transferrin.

**Figure 2.**
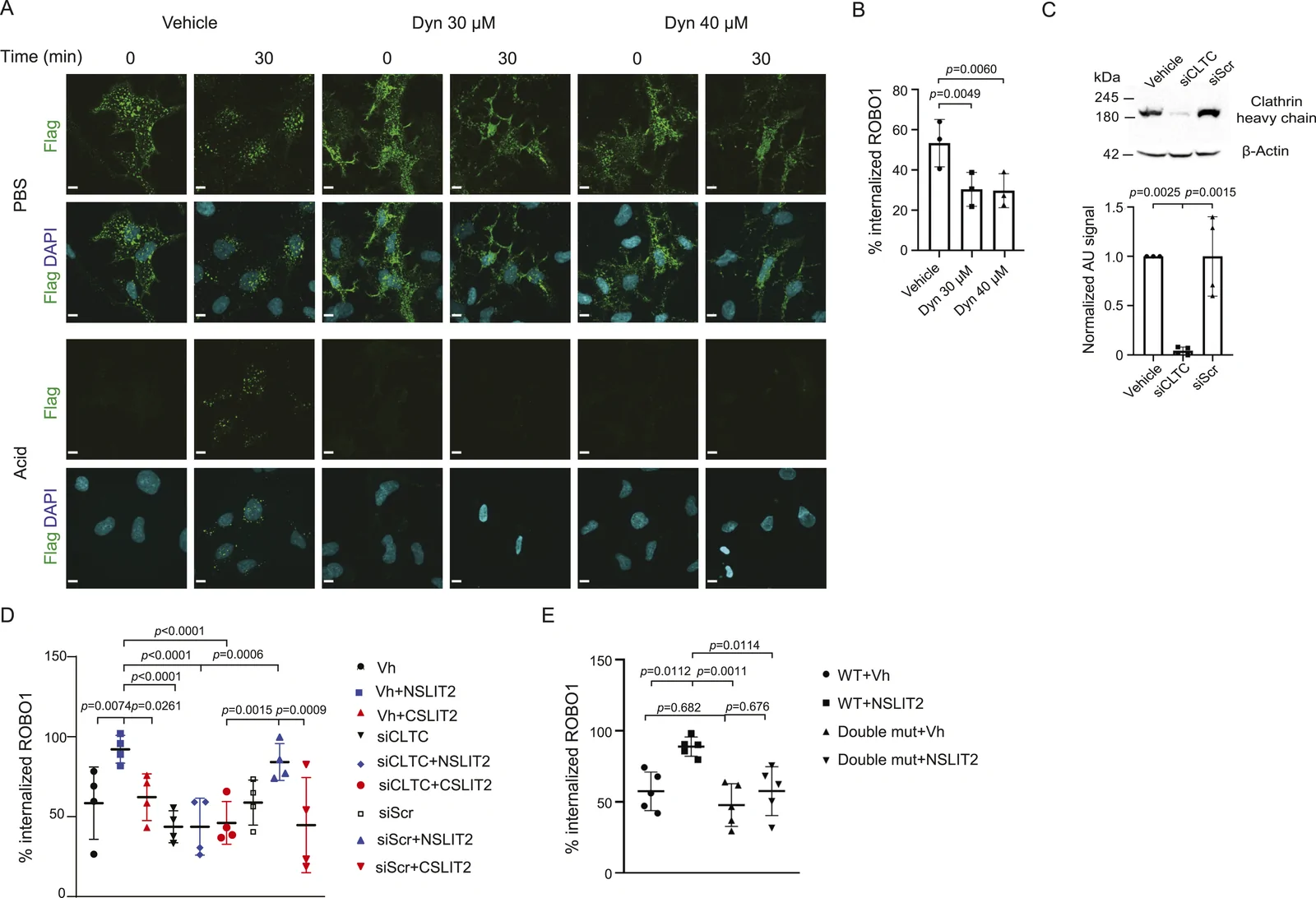
NSLIT2-induced endocytosis of ROBO1 is clathrin-dependent. **(A)** 5H9 cells were incubated with the dynamin inhibitor, Dyngo-4a (Dyn), at the concentrations indicated and endocytosis of ROBO1 assessed as in Fig 1A. Representative images are shown at the indicated time points. **(B)** Experiments were performed as in (A), and the percentage of ROBO1 internalized at 0 and 30 min was measured. Results are from three independent experiments. *P*-values were determined using two-way ANOVA followed by Sidak’s post hoc multiple comparison test. **(C)** Cells were incubated with specific siRNA targeting *CLTC* (siCLTC) or with scrambled, nontargeting siRNA (siScr). After 4 d, cell lysates were obtained and immunoblotting was performed to assess levels of clathrin. Representative images are shown. Quantification was from three independent experiments, and *P*-values were determined using one-way ANOVA followed by Tukey’s post hoc multiple comparison test. **(D)** Silencing of clathrin was performed as in (C) and endocytosis of ROBO1 assessed as in Fig 1B. Results are from four independent experiments. *P*-values were determined using two-way ANOVA followed by Sidak’s post hoc multiple comparison test. **(E)** Allele of ROBO1 in which both AP2-binding motifs were mutated was expressed in cells, and endocytosis experiments were performed as in (D). Results are from five independent experiments. *P*-values were determined using two-way ANOVA followed by Sidak’s post hoc multiple comparison test. Scale bars, 10 μm.

In *Drosophila*, SLIT-induced endocytosis of ROBO and subsequent axonal repulsion have been shown to be dynamin- and clathrin-dependent, and to require adaptor protein 2 (AP2)–binding motifs present in the cytoplasmic domain of ROBO1 (Chance & Bashaw, 2015). We therefore used Dyngo-4a, a potent dynamin inhibitor (McCluskey et al, 2013; Basagiannis et al, 2021), to investigate the role of dynamin in mediating endocytosis of ROBO1 in mammalian cells. As expected, inhibition of dynamin attenuated internalization of transferrin (Fig S2A and B). Dynamin inhibition decreased endocytosis of ROBO1 by 55% (Fig 2A and B; *P* = 0.0049 for Dyngo-4a 30 μM, versus vehicle; *P* = 0.006 for Dyngo-4a 40 μM, versus vehicle).

To examine the role of clathrin in endocytosis of ROBO1 in mammalian cells, we used siRNA to knock down clathrin to less than 10% of the levels in vehicle-treated cells or in cells exposed to scrambled siRNA (Fig 2C and *P* = 0.0025 and 0.0015, respectively) and confirmed that knockdown of clathrin prevented endocytosis of transferrin (Fig S2C and D; *P* = 0.0003, siCLTC versus vehicle, *P* = 0.0117, siCLTC versus siScr). In clathrin-deficient cells, NSLIT2-induced endocytosis of ROBO1 was markedly lower than in cells exposed to control vehicle or to scrambled siRNA (Fig 2D and *P* < 0.0001 versus vehicle; *P* = 0.0006 versus siScr).

Similar to ROBO in *Drosophila* (Chance & Bashaw, 2015), we found that human ROBO1 also contains two AP2-binding motifs (Yxxφ) (Collins et al, 2002) at the C terminus between the CC2 and CC3 domains of the protein (1311YGYI1314 and 1413YAGL1416). To examine the role of these AP2-binding motifs in endocytosis of mammalian ROBO1, we established HEK293A cells stably expressing mutant alleles of both AP2-binding sites (1311AGYA1314 and 1413AAGA1416). Exposure of cells expressing wild-type (WT) ROBO1 to NSLIT2 enhanced endocytosis by 36% (Fig 2E and *P* = 0.0112), whereas this effect was abolished in cells expressing ROBO1 harboring mutations in both AP2-binding sites (Fig 2E and *P* = 0.682, WT versus AP2-binding site mutant; *P* = 0.0114, WT + NSLIT2 versus AP2-binding site mut + NSLIT2). These results demonstrate that in mammalian cells, as in *Drosophila* (Chance & Bashaw, 2015), SLIT2-enhanced endocytosis of ROBO1 is dependent on dynamin, clathrin, and AP2-binding motifs in the intracellular domain of ROBO1.

### Inhibition of cell spreading by NSLIT2 requires clathrin-mediated endocytosis of ROBO1

**Figure S3.**
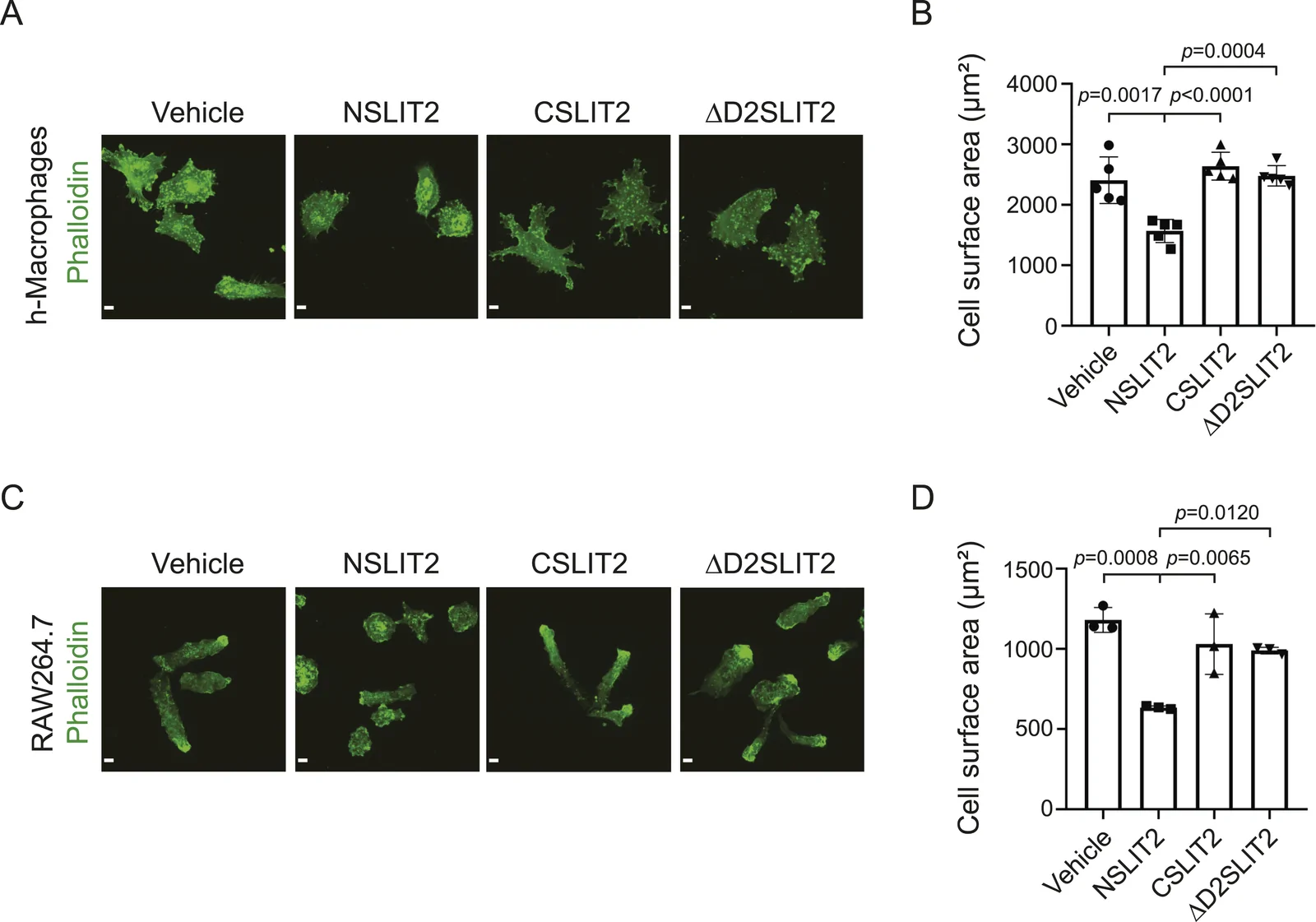
NSLIT2 inhibits spreading of human monocyte-derived macrophages and murine RAW 264.7 cells. **(A)** Experiments were performed as in Fig 3A using primary human monocyte-derived macrophages. Representative images are shown. **(B)** Experiments were performed as in Fig 3B. Results from five independent experiments were analyzed using one-way ANOVA followed by Tukey’s post hoc multiple comparison test. **(C)** Experiments were performed as in Fig 3A using RAW 264.7 cells. Representative images are shown. **(D)** Experiments were performed as in Fig 3B. Results from three independent experiments were analyzed using one-way ANOVA followed by Tukey’s post hoc multiple comparison test. Scale bars in images are 10 μm.

**Figure 3.**
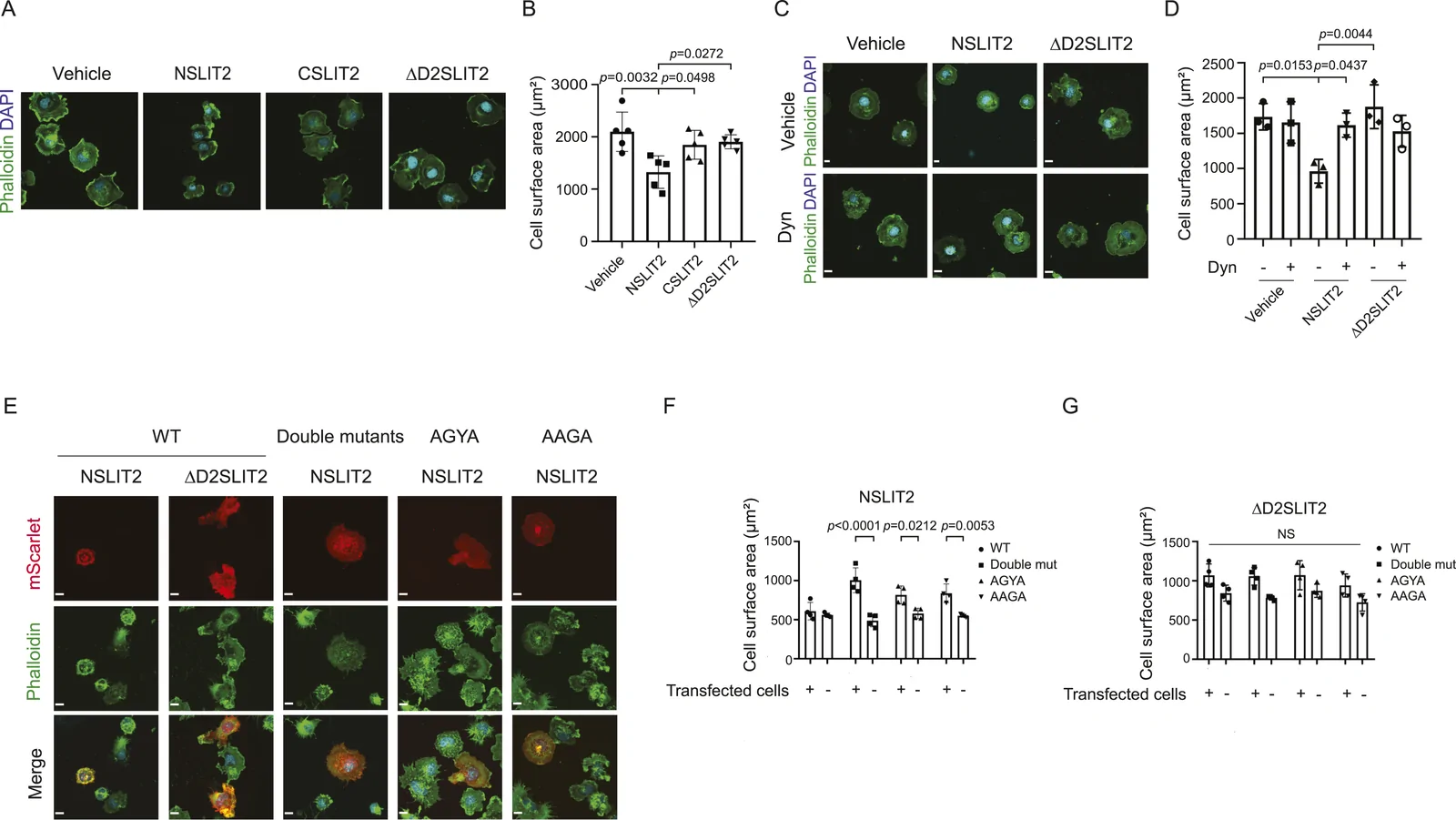
Clathrin-mediated endocytosis of ROBO1 is required for NSLIT2-induced inhibition of cell spreading. **(A)** COS-7 cells were incubated with NSLIT2, CSLIT2, or ΔD2SLIT2 for 15 min at 37°C, added onto poly-D-lysine–coated coverslips, and allowed to spread for 1 h. Cells were fixed, permeabilized, and incubated with AF488-conjugated phalloidin (green). **(B)** Experiments were performed as in (A), and cell surface area was measured. Results are from five independent experiments. *P*-values were determined using one-way ANOVA followed by Tukey’s post hoc multiple comparison test. **(C)** Cells were preincubated with the dynamin inhibitor, Dyngo-4a (25 μM), for 30 min and experiments performed as in (A). Representative images are shown. **(D)** Experiments were performed as in (C), and cell surface area was measured. Results are from three independent experiments. Results among groups were compared using two-way ANOVA followed by Sidak’s post hoc multiple comparison test. **(E)** Cells were transfected with plasmids encoding cmScarlet-ROBO1 FL or ROBO1 containing mutations of AP2-binding regions (AGYA, AAGA, double mutants). After 24 h, cell spreading assays were performed as in (A), incubating cells with NSLIT2 or ΔD2SLIT2. Representative images are shown with transfected cells indicated (in red). Scale bar, 10 μm. **(F)**. Spreading of untransfected cells (−), and cells transfected with ROBO1 plasmids (+) from experiments performed in (E) was measured after NSLIT2 treatment. **(G)** Spreading of untransfected cells (−), and cells transfected with ROBO1 plasmids (+) from experiments performed in (E) was measured after ΔD2SLIT2 treatment. Results are from four independent experiments. Results among groups were compared using two-way ANOVA followed by Sidak’s post hoc multiple comparison test.

We and others have previously shown that NSLIT2 inhibits spreading of diverse cell types, including neutrophils, platelets, and human and murine macrophages (Tole et al, 2009; Patel et al, 2012; Bhosle et al, 2020). We first confirmed that in primary human monocyte-derived macrophages and the murine RAW264.7 macrophage cell line, exposure of cells to NSLIT2 significantly decreased cell spreading, and that this response was not observed after exposure of cells to bio-inactive CSLIT2 or ΔD2SLIT2, which lacks the ROBO1-binding domain (Bhosle et al, 2020; Fig S3A–D; human macrophages: *P* < 0.0001 versus CSLIT2, *P* = 0.0004 versus ΔD2SLIT2; RAW264.7: *P* = 0.0065 versus CSLIT2, *P* = 0.0120 versus ΔD2SLIT2). We similarly observed that NSLIT2 significantly inhibited cell spreading of COS-7 cells (Fig 3A and B, *P* = 0.0498 versus CSLIT2, *P* = 0.0272 versus ΔD2SLIT2). To investigate the role of dynamin in NSLIT2-induced inhibition of cell spreading, we incubated COS-7 cells with the dynamin inhibitor, Dyngo-4a, and observed that NSLIT2 was no longer able to inhibit cell spreading (Fig 3C and D; *P* = 0.0437). In cells expressing either or both mutant alleles of the AP2-binding site, NSLIT2 failed to inhibit cell spreading, and the effect was most pronounced in cells expressing both mutant alleles (Fig 3E–G; *P* = 0.0212 versus AGYA, *P* = 0.0053 versus AAGA, *P* < 0.0001 versus double mutant). Taken together, these results suggest that in mammalian cells, NSLIT2-induced effects on cell morphology require clathrin-mediated endocytosis.

### Exposure of cells to NSLIT2 differentially regulates protein interactions with ROBO1

To elucidate the mechanism by which NSLIT2 affects cell morphology through clathrin-mediated endocytosis, we sought proteins differentially associated with ROBO1 by performing BioID expressing the full-length cBirA ROBO1 protein in HEK293 Flp-In cells, in the presence and absence of NSLIT2. Cells expressing a form of ROBO1 lacking the cytoplasmic C-terminal region (cBirA ROBO1 NTM) (Fig S1C) or the BirA-FLAG tag alone were used as controls.

Through the BioID analysis, we identified a number of proteins that were associated with full-length ROBO1 (ROBO1 FL) but not with ROBO1 lacking the intracellular domain (ROBO1 NTM) (Table 1). Among these proteins, we focused on those that are associated with ROBO1 FL under basal conditions, but not after exposure of cells to NSLIT2. These proteins included srGAP2, a known effector of SLIT-ROBO signaling (Wong et al, 2001; Guerrier et al, 2009; Charrier et al, 2012), and two of the five components of the WAVE complex, namely, NCKAP1 (NAP1) and CYFIP1 (Sra1), previously shown to interact with ROBO1 to modulate cytoskeletal remodeling (Table 1; Chen et al, 2014; Chaudhari et al, 2021). Interrogation of the BioID data also revealed around 30 candidate proteins that were associated with ROBO1 FL under basal conditions but no longer associated with ROBO1 FL after cell exposure to NSLIT2 (Table S3). None of the candidate proteins interacted with the form of ROBO1 lacking the intracellular domain (ROBO1 NTM) (Table S3). These data indicate that candidate proteins identified associate with the intracellular domain of ROBO1 under basal conditions and that exposure to NSLIT2 reverses this association. This list of candidate proteins includes NCKAP1 and CYFIP1, as well as other proteins associated with actin cytoskeletal remodeling, such as DLG5, MAP4K4, PHACTR4, RDX, PAK4, FARP1, MARK3, and NCK1 (Table 1; Matsumine et al, 1996; Li et al, 2001; Allen et al, 2004; Morales et al, 2004; Yang & Bashaw, 2006; Cheadle & Biederer, 2012; Liu et al, 2014; Vitorino et al, 2015; Gao et al, 2016; Sandí et al, 2017; Won et al, 2019). Unexpectedly, we detected a significant association between the intracellular domain of ROBO1 and APC, and observed that this interaction disappeared after exposure of cells to NSLIT2 (Table 1).

**Table 1.** Exposure to NSLIT2 differentially regulates protein interactions with ROBO1.

Gene symbol	Gene name	ROBO1_WT-BirA*-Flag								ROBO1_NTM-BirA*-Flag							
		(−) NSLIT2				(+) NSLIT2				(−) NSLIT2				(+) NSLIT2			
		Rep1	Rep2	SUM	BFDR	Rep1	Rep2	SUM	BFDR	Rep1	Rep2	SUM	BFDR	Rep1	Rep2	SUM	BFDR
ROBO1	*Roundabout guidance receptor 1*	1806	1877	3683	NA	1164	1153	2317	NA	479	488	967	NA	506	511	1017	NA
BirA	*Escherichia coli biotin ligase*	1813	2130	3943	NA	977	964	1941	NA	452	446	898	NA	451	480	931	NA
DLG5	*Discs, large homolog 5 (Drosophila)*	27	24	51	≤0.01	0	0	0	>0.01	0	0	0	>0.01	0	0	0	>0.01
CYFIP1	*Cytoplasmic FMR1 interacting protein 1 (Sra)*	19	16	35	≤0.01	0	0	0	>0.01	0	0	0	>0.01	0	0	0	>0.01
APC	*Adenomatous polyposis coli*	13	18	31	≤0.01	0	0	0	>0.01	0	0	0	>0.01	0	0	0	>0.01
MAP4K4	*Mitogen-activated protein kinase kinase kinase kinase 4*	10	15	25	≤0.01	0	0	0	>0.01	0	0	0	>0.01	0	0	0	>0.01
PHACTR4	*Phosphatase and actin regulator 4*	10	6	16	≤0.01	0	0	0	>0.01	0	0	0	>0.01	0	0	0	>0.01
RDX	*Radixin*	7	6	13	≤0.01	0	0	0	>0.01	0	0	0	>0.01	0	0	0	>0.01
NCKAP1	*NCK-associated protein 1 (NAP1)*	17	21	38	≤0.01	0	4	4	>0.01	0	0	0	>0.01	0	0	0	>0.01
PAK4	*p21 protein (Cdc42/Rac)-activated kinase 4*	4	3	7	≤0.01	0	0	0	>0.01	0	0	0	>0.01	0	0	0	>0.01
MARK3	*MAP/microtubule affinity-regulating kinase 3*	3	2	5	≤0.01	0	0	0	>0.01	0	0	0	>0.01	0	0	0	>0.01
FARP1	*FERM, RhoGEF (ARHGEF)*, *and pleckstrin domain protein 1 (chondrocyte-derived)*	13	11	24	≤0.01	4	3	7	>0.01	0	0	0	>0.01	0	0	0	>0.01
srGAP2	*SLIT-ROBO Rho GTPase*–*activating protein 2*	15	13	28	≤0.01	5	4	9	>0.01	0	0	0	>0.01	0	0	0	>0.01

A short list of statistically significant (BFDR ≤ 0.01) proximity interacting proteins of full-length, WT ROBO1 identified from the BioID performed in HEK293-T-REx Flp-In cells is shown. Values for each protein across different samples represent total spectral matches of assigned peptides. BFDR was calculated by comparing the spectral counts of each protein in the bait samples with their counts in the negative control samples (BirA* FLAG). HEK293 Flp-In cells expressing cBirA ROBO1 were incubated with NSLIT2 or vehicle control for 24 h together with tetracycline, and the results were compared with samples from cells expressing a form of ROBO1 lacking the cytoplasmic C-terminal region (cBirA ROBO1 NTM).

BFDR, Bayesian false discovery rate.

As expected, the proteins associated with ROBO1 lacking the cytoplasmic C terminus displayed little overlap with the ROBO1 FL, yielding only proximity interactions with plasma membrane, ER, and vacuolar membrane proteins not observed in the ROBO1 FL interactome.

Together, these results provide a human ROBO1 BioID interactome. Consistent with increased intracellular trafficking, this interactome changes in response to exposure to NSLIT2.

### ROBO1 is in close proximity to APC, and exposure to NSLIT2 disrupts this proximity

**Figure 4.**
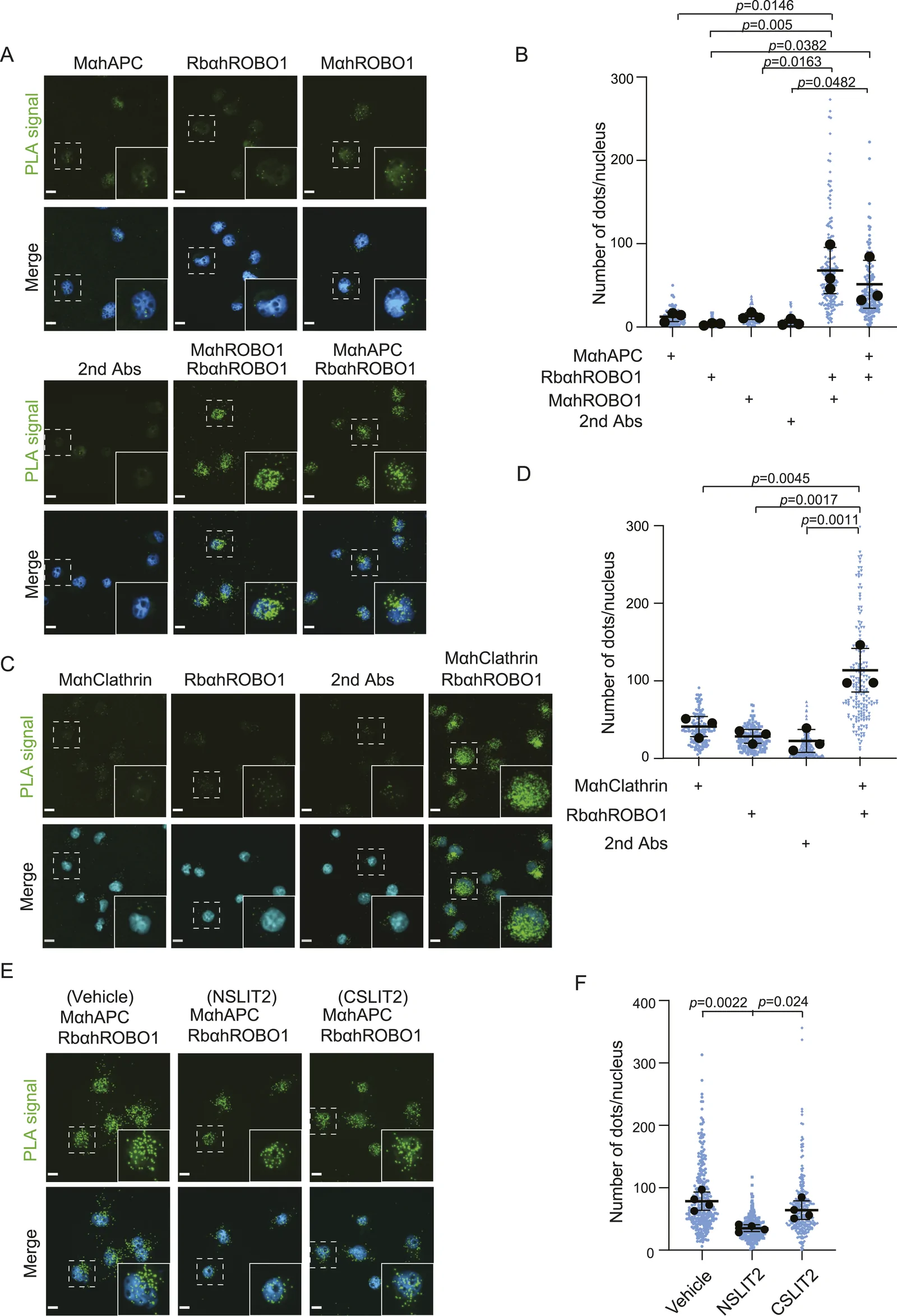
ROBO1 is in close proximity to APC and clathrin. **(A)** To detect proximity between ROBO1 and APC, PLAs were performed by incubating COS-7 cells with mouse anti-ROBO1 antibody recognizing the N-terminal region of ROBO1 (MAB7118; R&D Systems), rabbit anti-ROBO1 antibody recognizing the C-terminal region of ROBO1 (HPA052968; Sigma-Aldrich), mouse anti-APC antibody, and corresponding secondary antibodies. Representative images are shown. **(B)** Experiments were performed as in (A), and the number of dots per nucleus was determined. Individual data points are shown with the average from each experiment superimposed. Results are from three independent experiments. **(C)** To detect interaction between ROBO1 and clathrin, PLAs were performed using antibodies directed to ROBO1 (HPA052968; Sigma-Aldrich) and clathrin (X22, ab2731; Abcam Inc.). Representative images are shown. **(D)** Experiments were performed as in (C), and the number of dots per nucleus was determined. Individual data points are shown with the average from each experiment superimposed. **(E)** Cells were incubated with NSLIT2 or CSLIT2 and PLAs performed as in (A). Representative images are shown. **(F)** Experiments were performed as in (E), and the number of dots per nucleus was determined. Individual data points are shown with the average from each experiment superimposed. Results from four independent experiments were analyzed using one-way ANOVA followed by Tukey’s post hoc multiple comparison test. Scale bars in images are 10 μm.

**Figure S4.**
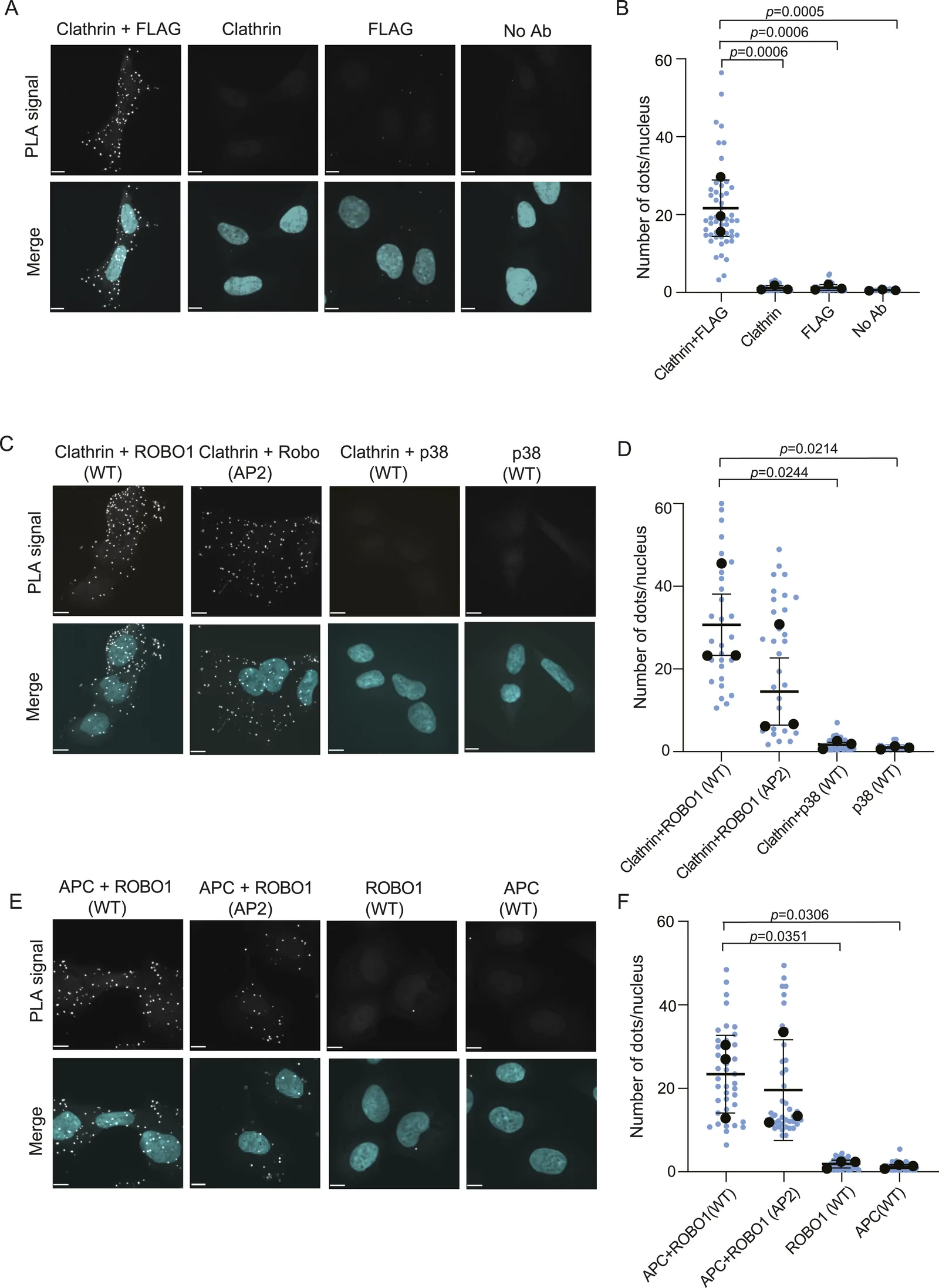
ROBO1 is in close proximity to clathrin. **(A)** PLA was performed in 5H9 cells (WT) using mouse anti-clathrin (ab2731; Abcam) and rabbit anti-FLAG antibodies (#2368; Cell Signaling Technology). Representative images are shown. **(B)** Experiments were performed as in (A), and the number of dots per nucleus was determined. Individual data points are shown with the average from each experiment superimposed. Results from three independent experiments were analyzed using one-way ANOVA followed by Tukey’s post hoc multiple comparison test. **(C)** PLA was performed in 5H9 cells (WT) or HEK293A cells stably expressing both AP2-binding site mutations (AP2), using mouse anti-clathrin (ab2731; Abcam), rabbit anti-FLAG (#2368; Cell Signaling Technology), and anti-p38 MAPK (#9212; Cell Signaling Technology). Representative images are shown. **(D)** Experiments were performed as in (C), and the number of dots per nucleus was determined. Individual data points are shown with the average from each experiment superimposed. Results from three independent experiments were analyzed using one-way ANOVA followed by Tukey’s post hoc multiple comparison test. **(E)** PLA was performed in 5H9 cells (WT) or HEK293A cells stably expressing both AP2-binding site mutations (AP2) using mouse anti-ROBO1 (MAB7118; R &D Systems) and anti-APC antibodies (ab40778; Abcam). Representative images are shown. **(F)** Experiments were performed as in (E), and the number of dots per nucleus was determined. Individual data points are shown with the average from each experiment superimposed. Results from three independent experiments were analyzed using one-way ANOVA followed by Tukey’s post hoc multiple comparison test. Scale bars in images are 10 μm.

We used proximity ligation assay (PLA), a highly sensitive and powerful technique for assessing the proximity of two proteins within 40 nm of each other (Schweitzer et al, 2000; Söderberg et al, 2006; Weibrecht et al, 2010; Gauthier et al, 2015). To validate the assay, we first confirmed the close proximity among ROBO1 receptors by labeling with both mouse- and rabbit-derived antibodies directed to human ROBO1 (Fig 4A and B). We next detected significant close proximity between ROBO1 and clathrin in COS-7 cells, in keeping with previous reports that ROBO1 undergoes CME (Fig 4C and D, *P* = 0.0011). We also detected significant close proximity between ROBO1 and APC in unstimulated COS-7 cells (Fig 4A and B, *P* = 0.0146). Importantly, this proximity was disrupted by exposure to NSLIT2, but not to bio-inactive CSLIT2 (Fig 4E and F; *P* = 0.0022, NSLIT2 versus vehicle; *P* = 0.024, NSLIT2 versus CSLIT2). The proximity between clathrin and ROBO1 was also confirmed in 5H9 cells (Fig S4A and B, *P* = 0.0005). As a further control, we examined proximity between clathrin and the cytosolic kinase, p38 MAPK. Clathrin and p38 MAPK did not exhibit significant proximity in the PLA (Fig S4C and D). This observation is consistent with the previous work demonstrating that β-arrestin scaffolds MAPK signaling complexes at clathrin-coated structures supporting MAPK signaling without requiring a direct interaction between p38 MAPK and clathrin (DeWire et al, 2007; Eichel et al, 2016).

The proximity of APC and ROBO1 was confirmed in 5H9 cells, expressing WT ROBO1 (Fig S4E and F, *P* = 0.0306). PLA signals were also detected in HEK293 cells stably expressing ROBO1 with mutations in both AP2-binding motifs (Fig S4E and F). However, because the anti-ROBO1 and anti-APC antibodies recognize both endogenous and exogenous proteins, the observed PLA signals likely reflect a combination of endogenous and exogenous protein interactions. Therefore, although these findings further support the proximity between ROBO1 and APC, consistent with our observations in COS-7 cells (Fig 4), they do not allow us to conclusively determine whether mutation of the AP2-binding motifs affects the interaction between ROBO1 and APC. Interestingly, in HEK293 cells stably expressing ROBO1 containing mutations of both AP2-binding motifs, the proximity between ROBO1 and clathrin was not significantly reduced (Fig S4C and D). These findings suggest that the proximity between ROBO1 and clathrin is not solely dependent on the AP2-binding motifs and may involve additional adaptor proteins or alternative mechanisms.

Collectively, these results support a model in which ROBO1, APC, and clathrin exist within a closely associated signaling complex, potentially localized to clathrin-coated pits. Exposure to NSLIT2 promotes dissociation of APC from ROBO1, which may facilitate ROBO1 internalization through clathrin-mediated endocytosis.

### APC modulates NSLIT2-induced endocytosis of ROBO1

**Figure 5.**
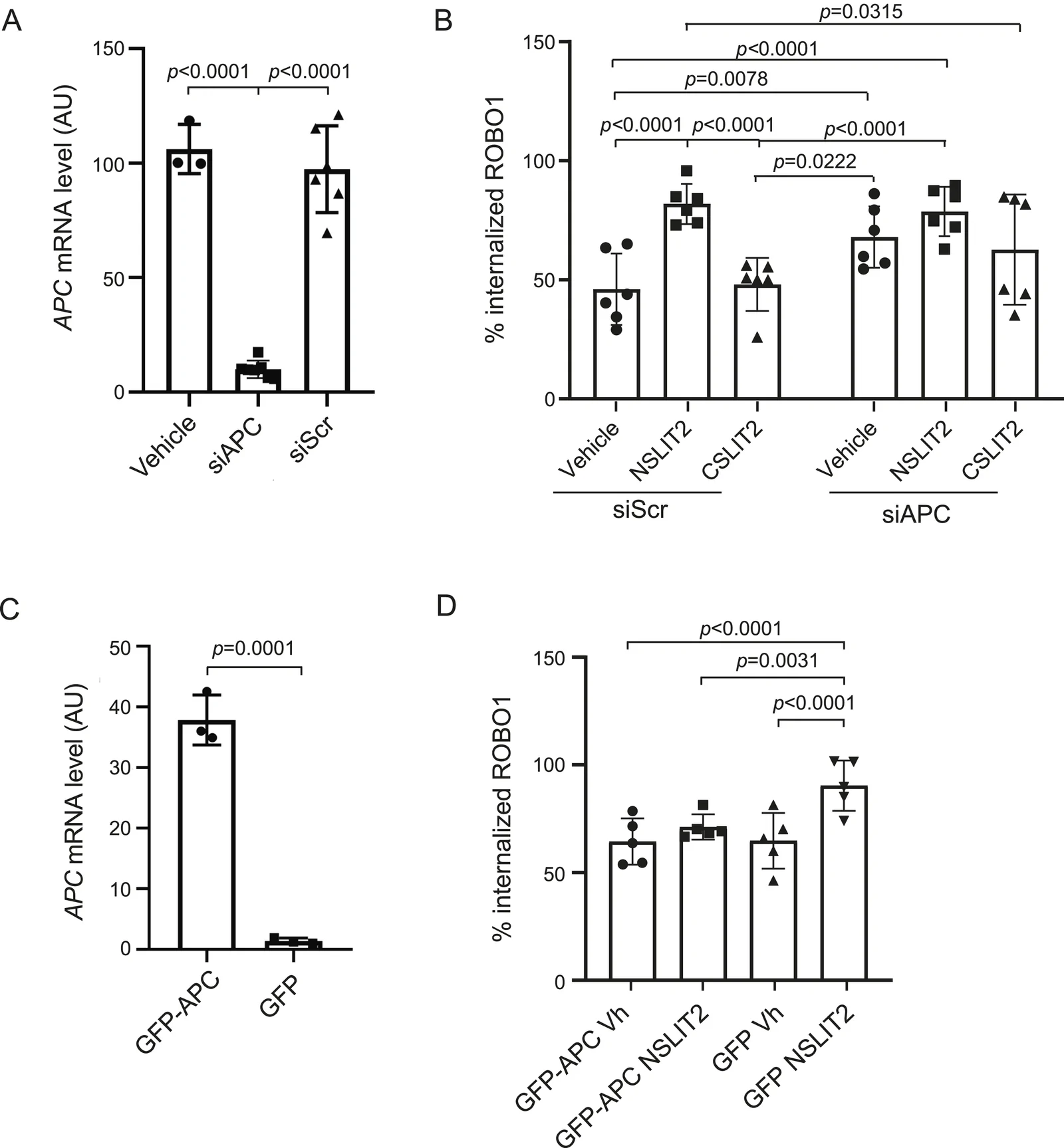
APC modulates NSLIT2-induced endocytosis of ROBO1. **(A)** 5H9 cells were transfected with siRNA specifically targeting *APC* (siAPC) or with scrambled siRNA (siScr). After 4 d, RNA was extracted and *APC* expression was measured using qRT-PCR. Results from 3 to 5 independent experiments were analyzed using one-way ANOVA followed by Tukey’s post hoc multiple comparison test. **(B)** *APC* was silenced as in (A), and endocytosis assays were performed as in Fig 1B. Results from six independent experiments were analyzed using two-way ANOVA followed by Sidak’s post hoc multiple comparison test. **(C)** 5H9 cells were stably transfected to express GFP-APC or GFP alone. RNA was extracted and *APC* expression measured using qRT-PCR. Results from three independent experiments were analyzed using a two-tailed paired *t* test. **(D)** ROBO1 endocytosis assay was performed as shown in (C). The plot was generated from five independent experiments. Experiments were analyzed using two-way ANOVA followed by Sidak’s post hoc multiple comparison test. GFP-APC, GFP-tagged APC.

To investigate the role of APC in the endocytosis of ROBO1, we used siRNA to knock down APC in 5H9 cells to less than 10% (Fig 5A). In cells incubated with nontargeting scrambled siRNA, exposure to NSLIT2 resulted in significantly more internalization of ROBO1 than in cells incubated with vehicle or with bio-inactive CSLIT2 (Fig 5B and *P* < 0.0001, NSLIT2 versus vehicle; *P* < 0.0001, NSLIT2 versus CSLIT2). In cells deficient in APC, endocytosis was similar in the presence and absence of NSLIT2 (Fig 5B). Importantly, APC depletion significantly increased ROBO1 internalization under basal conditions (Fig 5B and *P* = 0.0078, siScr versus siAPC), consistent with the expectation that APC depletion would, at least in part, mimic the effect of NSLIT2-induced APC dissociation. Together, these findings indicate that APC depletion increases constitutive ROBO1 endocytosis and eliminates the further stimulatory effect of NSLIT2.

In cells overexpressing APC by ∼30-fold (Fig 5C and *P* = 0.0001), the basal level of endocytosis of ROBO1 was similar to that of cells expressing normal levels of APC (Fig 5D and *P* = 0.7539). Exposure of such APC-overexpressing cells to NSLIT2 failed to stimulate endocytosis, in contrast to the NSLIT2-enhanced endocytosis seen in cells with normal levels of APC (Fig 5D and *P* = 0.0031). Together, these results suggest that APC is an endogenous inhibitor of endocytosis for ROBO1 at the cell surface.

### APC is necessary for NSLIT2-induced inhibition of cell spreading

**Figure 6.**
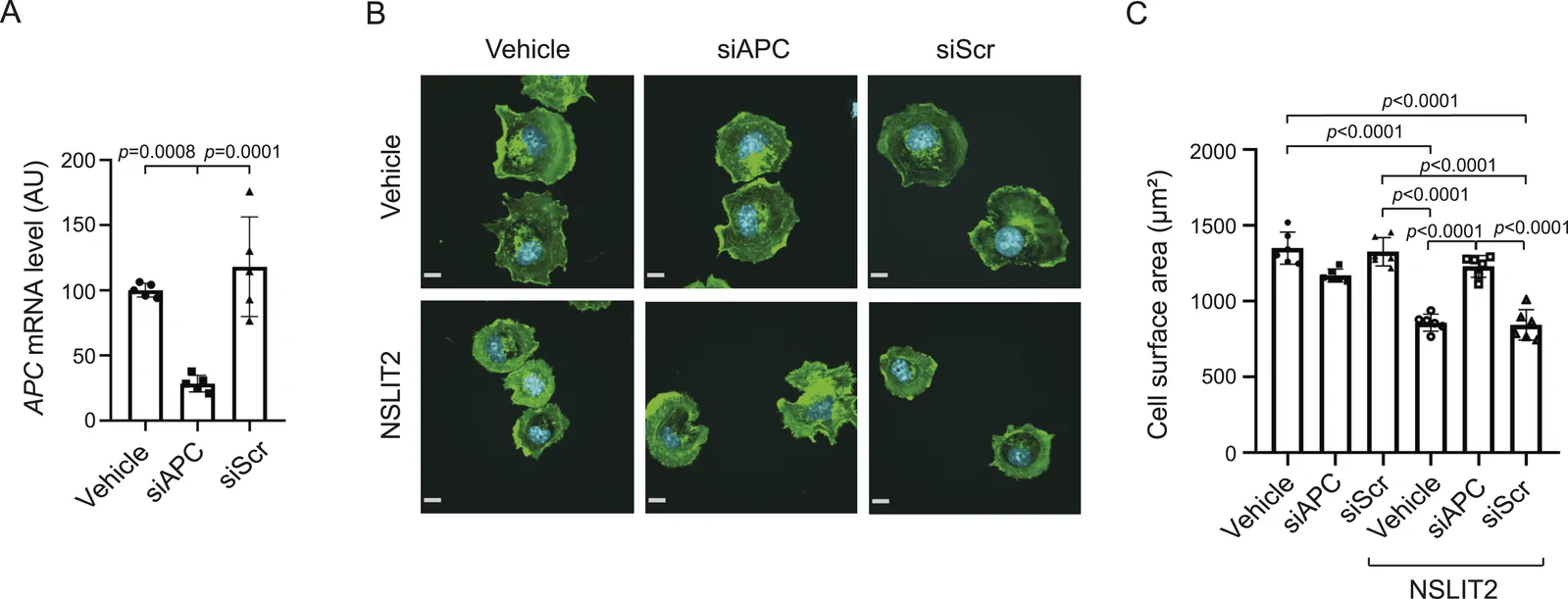
APC is required for NSLIT2-induced inhibition of cell spreading. **(A)** COS-7 cells were transfected with siRNA targeting *APC* (siAPC) or with scrambled siRNA (siScr). After 4 d, RNA was extracted and *APC* expression assessed using qRT-PCR. Results from five independent experiments were analyzed using one-way ANOVA followed by Tukey’s post hoc multiple comparison test. **(B)** COS-7 cells were transfected with siRNA targeting *APC* (siAPC) or with scrambled siRNA (siScr). After 4 d, cell spreading assays were performed as in Fig 3A. Representative images are shown. Scale bars are 10 μm. **(C)** Experiments were performed as in (B). Results from five independent experiments were analyzed using one-way ANOVA followed by Tukey’s post hoc multiple comparison test.

Having observed that APC inhibits NSLIT2-induced endocytosis of ROBO1, we questioned whether APC is required for the inhibitory effect of NSLIT2 on cell spreading. In COS-7 cells, as in 5H9 cells, we found that a targeted siRNA reduced levels of APC to 25–30% of the levels detected in vehicle-treated cells or in cells exposed to nontargeting scrambled siRNA (Fig 6A). In cells with reduced APC, NSLIT2 was no longer able to inhibit cell spreading compared with vehicle-treated cells and cells exposed to scrambled siRNA (Fig 6B and C; *P* < 0.0001). These findings indicate that increased constitutive ROBO1 endocytosis resulting from APC depletion is not sufficient to reproduce the effect of NSLIT2 on cell spreading. Conversely, the ability of NSLIT2 to inhibit cell spreading requires APC, suggesting that this effect is not simply a consequence of increasing the overall amount of ROBO1 internalization. Rather, APC may be required for NSLIT2 to regulate the internalization of a specific pool of ROBO1 or to control the spatial or temporal pattern of ROBO1 internalization at the cell surface. Such regulated endocytosis may be necessary to engage the downstream signaling or cytoskeletal changes that inhibit cell spreading.

**Figure S5.**
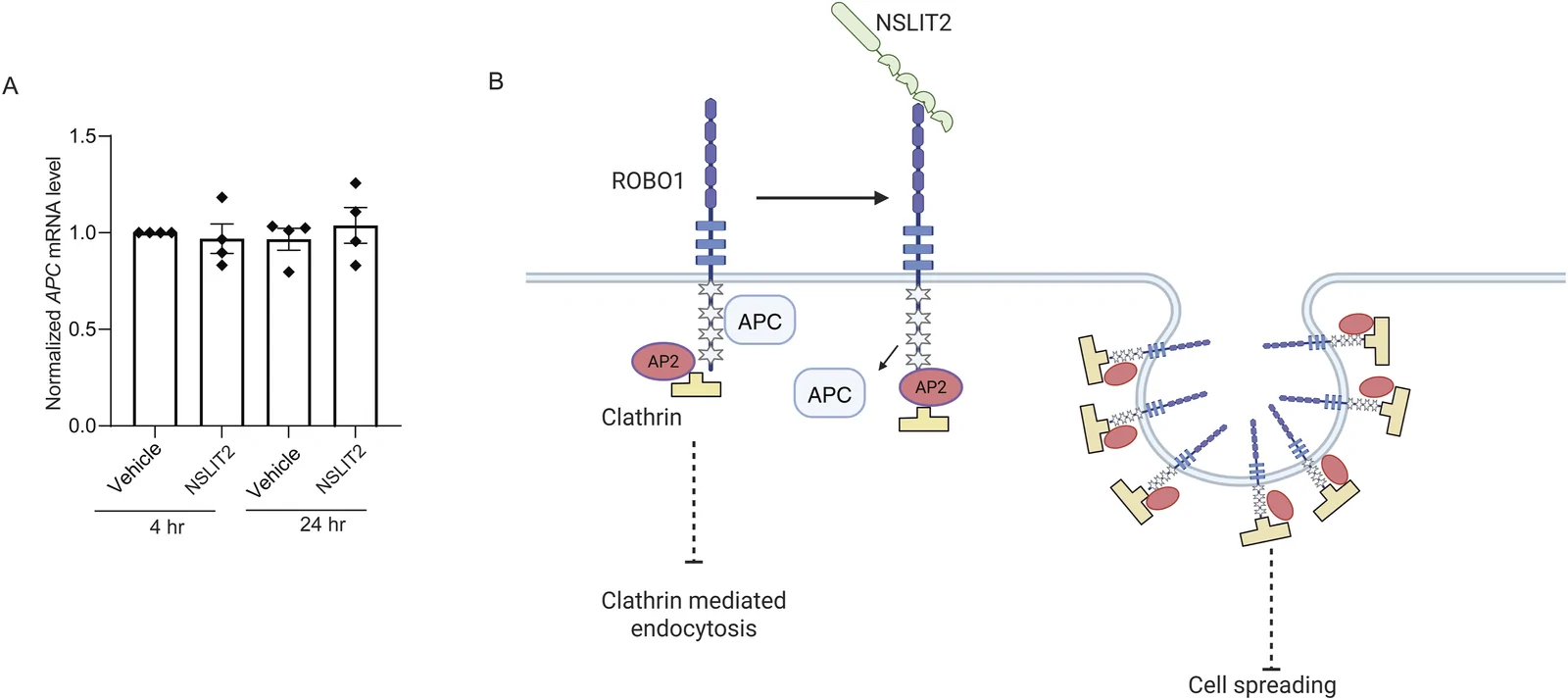
NSLIT2 does not affect APC mRNA expression. **(A)** 5H9 cells were incubated with 30 nM NSLIT2 or PBS for 4 and 24 h at 37°C. RNA was then extracted, and *APC* expression was measured using qPCR. Results from four independent experiments were analyzed using one-way ANOVA followed by Tukey’s post hoc multiple comparison test. **(B)** Schematic depicting the proximities of ROBO1, APC, and clathrin. A pool of ROBO1 exists in close proximity to APC, AP2, and clathrin. Upon binding of NSLIT2 to ROBO1, APC disengages from the intracellular domain of ROBO1, allowing ROBO1 to undergo clathrin-mediated endocytosis and subsequently inhibiting cytoskeletal rearrangements. Created in BioRender. Lab, G. (2026) https://BioRender.com/m5ia1pq.

To assess whether NSLIT2 regulates *APC* expression at the transcriptional level, we performed quantitative polymerase chain reaction (qRT-PCR) analysis of *APC* mRNA after exposure of cells to NSLIT2 for 4 and 24 h. No significant changes in *APC* mRNA expression were observed at either time point (Fig S5A). Previous studies have shown that SLIT2-ROBO signaling primarily mediates rapid posttranslational regulation of protein interactions and cytoskeletal dynamics rather than transcriptional regulation (Blockus & Chédotal, 2016; Bhosle et al, 2020). Consistent with this mechanism, our findings suggest that NSLIT2 influences APC function through modulation of protein–protein interactions, including the APC-ROBO1 association, rather than through changes in *APC* transcript abundance. Taken together, these results suggest that APC acts as an endogenous inhibitor of endocytosis of a distinct pool of cell surface ROBO1 that is sensitive to NSLIT2 and is required for NSLIT2-mediated inhibition of cell spreading (Fig S5B).

## Discussion

Endocytosis is an essential process that allows cells to regulate the levels of receptors on their surface, control the signaling pathways activated by extracellular ligands, and internalize essential nutrients (Sorkin & von Zastrow, 2009; McMahon & Boucrot, 2011). ROBO receptors also undergo endocytosis. It has been reported in *Drosophila* that ROBO1 endocytosis is clathrin-dependent and is essential for receptor activation and proper midline axon repulsion by allowing recruitment of the downstream effector, Son of Sevenless (Chance & Bashaw, 2015). In mammalian cells, it has similarly been reported that endocytosis and recycling of ROBO1 are required for SLIT-induced responses to regulate midline crossing of commissural axons (Kinoshita-Kawada et al, 2019). Our data demonstrate that in a variety of mammalian cells, ROBO1 also undergoes NSLIT2-induced endocytosis that is dynamin- and clathrin-dependent and that CME is necessary for NSLIT2-induced inhibition of cell spreading. To further identify ROBO1-interacting proteins in an unbiased, sensitive, and specific manner, we used BioID, enabling us to detect both physiologically relevant and weak protein–protein interactions (Roux et al, 2012, 2018). Using BioID, we identified around 11 candidate proteins that interact with the cytoplasmic region of ROBO1 under basal conditions and dissociate from ROBO1 upon exposure of cells to NSLIT2 (Table 1). ROBO1-interacting proteins identified include important actin cytoskeletal regulatory proteins, among them two of the five components of the WAVE complex, namely, NCKAP1 (NAP1) and CYFIP1 (Sra) (Takenawa & Suetsugu, 2007; Chen et al, 2014). Unexpectedly, we noted that one of the strongest “hits” identified was APC, originally identified as an important tumor suppressor in the human colon and which is conserved across species (Aoki & Taketo, 2007). APC is a multidomain protein that contains binding sites for numerous proteins, including microtubules, the Wnt/Wg pathway components β-catenin and axin, the cytoskeletal regulators EB1 and IQGAP1, and the Rac guanine nucleotide exchange factor Asef1 (Kawasaki et al, 2000; Watanabe et al, 2004; Aoki & Taketo, 2007; Hedman et al, 2015). Activation of the canonical Wnt signal plays an essential role in tumorigenesis caused by APC mutations. APC plays additional roles in other cellular processes, such as cell migration, adhesion, and chromosome segregation (Kawasaki et al, 2000; Watanabe et al, 2004; Aoki & Taketo, 2007; Kroboth et al, 2007; Nelson & Näthke, 2013; Hedman et al, 2015). Interestingly, APC promotes axon guidance by integrating extracellular signals with neuronal polarity and cytoskeletal organization at the growing axon tip. Through local inhibition of glycogen synthase kinase-3β, APC accumulates at microtubule plus ends where it stabilizes microtubules and transports polarity proteins such as mPar3, thereby enabling directed axon growth (Shi et al, 2004; Zhou et al, 2004). An important avenue of further investigation would be to examine potential interactions of ROBO1 and other components of the WAVE complex, including NCKAP1 and CYFIP1, and whether SLIT2 affects the expression of these interactors.

Using PLA, we observed that ROBO1, APC, and clathrin are in close proximity to one another, suggesting that they may form a complex in clathrin-coated pits. This is in keeping with studies demonstrating that APC interacts with the AP2-clathrin complex (Saito-Diaz et al, 2018). We further noted that knockdown of APC resulted in more constitutive endocytosis compared with that seen in cells exposed to scrambled siRNA, suggesting that APC negatively regulates endocytosis. When cellular levels of APC were intact, exposure of cells to NSLIT2 resulted in significantly enhanced endocytosis, as well as reduced cell spreading, but these NSLIT2-induced effects were lost in cells lacking APC. These data are in keeping with reports that under basal conditions, APC exists in complex with clathrin to prevent constitutive, ligand-independent, clathrin-dependent signalosome formation and Wnt pathway activation (Saito-Diaz et al, 2018). We further observed that cells expressing overabundant amounts of APC undergo similar rates of endocytosis compared with cells expressing usual amounts of APC, but unlike in cells expressing normal levels of APC, exposure to NSLIT2 failed to enhance endocytosis in cells overexpressing APC. Together, these results suggest that a limited pool of APC associates with clathrin complexes to constitutively inhibit endocytosis, and that NSLIT2 causes disengagement of this pool of APC from ROBO1, thereby promoting increased internalization of ROBO1 (Fig S5B). These results are in keeping with crystallographic structural data demonstrating that binding of NSLIT2 to ROBO1 does not promote changes in oligomerization of ROBO1. Instead, it suggests that the binding of NSLIT2 induces a conformational change in ROBO1 that enables subsequent transduction of intracellular signals (Barak et al, 2019). We found that even in the absence of NSLIT2, ROBO1 undergoes some degree of constitutive endocytosis. It has been reported that TGF-β receptors undergo internalization through both clathrin-coated pits and caveola–lipid raft internalization pathways. Clathrin-dependent internalization into the early endosome promotes the activation of the TGF-β signaling cascade, whereas the caveolin-1–positive lipid raft compartment is required for receptor degradation (Di Guglielmo et al, 2003). Other authors reported that nerve growth factor leads to the redirection of a pool of p75 neurotrophin receptor (p75NTR) into clathrin-coated pits and internalization of p75NTR via clathrin-mediated endocytosis. However, exposure to nerve growth factor does not enhance the internalization or degradation of p75NTR, which undergoes a rapid dynamin-dependent and clathrin-independent recycling process in motor neurons (Deinhardt et al, 2007). Similarly, at low doses, EGF has been shown to direct its receptor, EGFR, to a clathrin-mediated pathway of internalization, resulting in activation of downstream targets. However, at high concentrations, EGF promotes EGFR uptake via a clathrin-independent mechanism, resulting in increased receptor degradation (Sigismund et al, 2005).

In keeping with observations from other groups, our findings suggest that ROBO1 may undergo clathrin-dependent and clathrin-independent endocytosis. A separate pool of ROBO1 is acutely responsive to NSLIT2, suggesting that in the basal state, this pool of ROBO1 may exist in complex with APC, AP2, and clathrin, inhibiting endocytosis of ROBO1. After binding of NSLIT2, ROBO1 may undergo conformational changes (Barak et al, 2019) that actively disengage APC from the ROBO1 intracellular domain (Fig S5B). An important avenue for future investigation will be to determine whether NSLIT2-induced APC relocalization influences cell migration by dissociating APC from its known regulatory binding partners, including the Rac-specific guanine nucleotide exchange factors Asef1 and Asef2 and/or IQGAP. Such a mechanism could modulate Rac1 and Cdc42 signaling, leading to inhibition of the actin cytoskeletal rearrangements required for cell spreading and polarized migration (Watanabe et al, 2004; Hedman et al, 2015). The clathrin-independent endocytosis pathway may distinctly participate in the recycling or degradation of ROBO1. Future studies should investigate how internalization through these two distinct pathways is differentially regulated for ROBO1 and other ROBO family members, particularly ROBO2, and determine whether NSLIT2 selectively affects distinct pools or isoform-specific trafficking and signaling mechanisms.

## Materials and Methods

### Antibodies and reagents

Primary or secondary antibodies were purchased from the following vendors: rabbit anti-human ROBO1 (PA5-29917; Thermo Fisher Scientific); rabbit anti-human ROBO1 (HPA052968; Sigma-Aldrich); mouse monoclonal anti-human ROBO1 (MAB7118; R&D Systems); mouse monoclonal anti-human APC (CC-1) (ab16794; Abcam Inc.); rabbit monoclonal anti-clathrin heavy chain (D3C6) (#4796; Cell Signaling Technology); mouse monoclonal anti-clathrin heavy chain (X22) (ab2731; Abcam Inc.); mouse monoclonal anti-β-actin (AC004; ABclonal); mouse monoclonal anti-FLAG (M2) (F1804; Sigma-Aldrich); peroxidase AffiniPure goat anti-mouse IgG (H + L) (115-035-003; Jackson ImmunoResearch Laboratories, Inc.); peroxidase AffiniPure goat anti-rabbit IgG (H + L) (111-035-144; Jackson ImmunoResearch Laboratories, Inc.); Alexa Fluor (AF) 488 AffiniPure Fab fragment goat anti-mouse IgG (115-547-003; Jackson ImmunoResearch Laboratories, Inc.). Detailed information about the antibodies used is provided in Table S1. AF488-conjugated transferrin was from Molecular Probes by Invitrogen Life Technologies (T13342; Thermo Fisher Scientific).

Table S1. Antibodies.

The ADAM10 inhibitor GI 254023X was from Sigma-Aldrich Canada (SML0789); the dynamin inhibitor Dyngo-4a was from Abcam (ab120689).

Proximity ligation kits were from Sigma-Aldrich: Duolink In Situ PLA Probe Anti-Rabbit PLUS, DUO92002; Duolink In Situ PLA Probe Anti-Mouse MINUS, DUO92004; Duolink In Situ Detection Reagents FarRed, DUO92013.

### DNA plasmid construction

In order to construct pcDNA5 FRT/TO ROBO1-WT-BirA*-FLAG with restriction sites AscI-NotI, human ROBO1 (NP_002932) (Fig S1A) was first subcloned from pYFP-C1 ROBO1 WT in pZeoSV2 vector with NheI and HindIII. A linker with NheI-AscI-EcoRV was added in-frame to the N terminus of the ROBO1 sequence. A ROBO1 C-terminal sequence starting from SalI was substituted by PCR to introduce a NotI restriction enzyme site upstream of HindIII. The ROBO1 full-length DNA sequence was then subcloned into the pcDNA5 FRT/TO-BirA*-FLAG plasmid with AscI-NotI restriction sites (Fig S1B). An N-terminal ROBO1 including the transmembrane domain (M1-R920) was PCR-amplified with AscI-NotI sites and subcloned into the pcDNA5 FRT/TO-BirA*-FLAG vector to make a pcDNA5 FRT/TO ROBO1-NTM-BirA*-FLAG construct (Fig S1C). pcDNA3.1zeo+/cmScarlet-hROBO1 FL was subcloned with NheI-HindIII, and mScarlet was PCR-cloned with HindIII-XhoI from pmScarlet C1 and subcloned in-frame into the pcDNA3.1zeo+ vector (Fig S1D). pcDNA3.1zeo+/nFLAG-cmScarlet-hROBO1 was generated by inserting the FLAG tag sequence at the N terminus after the signal peptide sequence of ROBO1 with QuikChange II Site-Directed Mutagenesis Kit (Agilent Technologies, Inc.). pcDNA3.1zeo+/nFLAG-intMyc-cmScarlet-hROBO1 was generated by inserting the Myc tag sequence at the extracellular juxtamembrane region of ROBO1, downstream of a predicted ADAM10 cleavage site (Q888-Q889), in the pcDNA3.1zeo+/nFLAG-cmScarlet-hROBO1 construct (Fig S1E). The two AP2-binding sites were modified singly or in combination by site-directed mutagenesis in the pcDNA3.1zeo+/cmScarlet-hROBO1 plasmid. Mutation of both AP2-binding sites was established by digesting the pcDNA3.1zeo+/cmScarlet-hROBO1-AP2-binding site double mutant construct with BamH1 and HindIII fragments spanning the two AP2-binding sites and substituting in pcDNA3.1zeo+/nFLAG-intMyc-cmScarlet-hROBO1 WT (Fig S1E and F). All DNA plasmid sequences were confirmed by automated sequencing. The pEGFP-C-APC full-length DNA plasmid was kindly provided by Dr. Angela I. M. Barth (Barth et al, 2002). All primer sequences for PCR cloning or qRT-PCR are provided in Table S2.

Table S2. Primers used for cDNA plasmid construction and qPCR experiments.

### Cell culture

The HEK293A, RAW 264.7, and COS-7 cell lines were obtained from and authenticated by the American Type Culture Collection. Cells were grown in RPMI-1640 medium containing 5% heat-inactivated FBS at 37°C with 5% CO_2_. As endogenous levels of ROBO1 are relatively low in COS-7 cells, we also generated a HEK293A cell line that stably expresses ROBO1. To generate stable cell lines expressing FL ROBO1, the pcDNAZeo+/nFLAG-intMyc-cmScarlet-ROBO1 FL plasmid (Fig S1E) was transfected in HEK293A cells, cultured, and selected in the presence of Zeocin for 2 wk. Cells were sorted by flow cytometry to select cells with red fluorescence, and a single cell clone (5H9) was obtained by the limited dilution method. HEK293A cells stably expressing the pcDNAZeo+/nFLAG-intMyc-cmScarlet-ROBO1 FL plasmid (Fig S1E) are hereafter referred to as 5H9 cells.

The DNA plasmid nFLAG-intMyc-cmScarlet ROBO1-AGYA-AAGA (Fig S1E and F) encoding ROBO1 containing point mutations of both AP2-binding sites was transfected in HEK293A cells. After cell sorting by flow cytometry and Zeocin selection, we obtained stable polyclonal cells for subsequent experiments.

Cells stably expressing APC and ROBO1 were generated by transfecting pEGFP-APC into 5H9 cells and were selected by culturing in the presence of G418; cell sorting by flow cytometry was performed to select cells demonstrating both green (APC) and red (ROBO1) fluorescence. For comparison, a cell line with GFP-C was established simultaneously.

293 T-Rex Flp-In cells stably expressing ROBO1-WT-BirA*-FLAG or ROBO1-NTM-BirA*-FLAG were generated using the Flp-In system (Invitrogen, Thermo Fisher Scientific) after hygromycin B selection (Coyaud et al, 2015).

Peripheral blood mononuclear cells were isolated from the blood of healthy volunteers by density gradient separation using Lympholyte-H (Cedarlane). The protocol (#1000070040) for human blood collection from healthy adult donors was reviewed and approved by The Hospital for Sick Children (SickKids) Research Ethics Board. Written, informed consent was obtained from all participants before their participation. The cells were cultured in two 10-cm dishes in RPMI-1640 medium supplemented with 10% heat-inactivated FBS, 1% penicillin/streptomycin/amphotericin B, and 10 ng/ml of human macrophage colony-stimulating factor for 7–12 d (Wong et al, 2016).

### siRNA gene silencing

siRNA siGENOME targeting human clathrin heavy chain (CLTC) was from Dharmacon (D-004001-02-0005); siRNA ON-TARGETplus human siRNA SMARTpools targeting human APC were purchased from Dharmacon (L-003869-00-0005); siGENOME nontargeting siRNA was from Dharmacon (D-001210-01-20). siRNA transfection was performed using reverse transfection protocols with Lipofectamine RNAiMAX transfection reagent (13778; Life Technologies). For CLTC knockdown, cells were transfected with specific siRNA (65 nM) and experiments performed 72 h later. For APC knockdown, cells were transfected with specific siRNA (50 nM) on Day 0 and Day 2 and experiments performed on Day 4 (96 h). The siRNA-mediated knockdown efficiency was confirmed by either immunoblotting or qRT-PCR.

### RNA isolation, reverse transcription, and qRT-PCR

Total RNA was isolated from cells using RNeasy Plus Mini Kit (74034; Qiagen), and reverse transcription was performed with 1–2 μg RNA using SuperScript IV VILO MasterMix (11756050; Thermo Fisher Scientific) with the following conditions: 25°C for 10 min, 50°C for 10 min, 85°C for 5 min, and products were stored at −20°C until qRT-PCR. qRT-PCR was performed using SYBR Green PCR Master Mix Kit (4367659; Applied Biosystems). Human GAPDH and green monkey β-actin were used as reference controls. qRT-PCR data were obtained using QuantStudio 3 Real-Time PCR System, and analysis was performed using Design and Analysis Software v2 (Applied Biosystems). All primer sequences for qRT-PCR are provided in Table S2.

### Endocytosis assay

Glass coverslips were precoated with poly-D-lysine (50 μg/ml; MW 70–150 D, P6407; Sigma-Aldrich) for 30 min at 37°C. Cells were grown in 24-well plates containing the coated glass coverslips. Cells were treated with ADAM10 inhibitor, GI-254023X, 10 μM, for 16 h to allow ROBO1 to accumulate on the cell surface. Cells were washed twice with PBS to remove the ADAM10 inhibitor and incubated for 30 min with fresh complete RPMI-1640 medium. Cells were incubated with mouse monoclonal anti-FLAG antibody (1.5 μg/ml) for 1 h at 4°C with slow rotation. The medium was removed, and cells were then incubated at 37°C with bioactive NSLIT2 (30 nM) or an equimolar concentration of bio-inactive CSLIT2 for different time periods. NSLIT2 contains the N-terminal fragment of SLIT2 and can bind the ROBO1 receptor. CSLIT2 contains the C-terminal fragment of SLIT2 and cannot bind to ROBO1 (Tole et al, 2009; Patel et al, 2012). Endocytosis was arrested by placing plates on ice, and anti-FLAG antibody binding to ROBO1 was removed from the cell surface using acid wash buffer (100 mM acetic acid, 0.5 M NaCl in PBS, pH 3.1) for 1 min followed by washing three times with PBS. A parallel sample was established for each time point, undergoing only three PBS washes to detect total ROBO1. Cells were fixed with 4% PFA and permeabilized with 0.1% Triton X-100 and 0.1 M glycine for 15 min each. Cells were blocked with 5% goat serum and labeled with AF488-Fab fragment goat anti-mouse IgG plus DAPI for 45 min. The percentage of internalized ROBO1 was calculated based on this equation (Fourgeaud et al, 2003):

$$\%\;internalized\left(t\right)=100x\frac{intracellular\left(t\right)+\left(surface\left(t_{0}\right)-total\left(t\right)\right)}{surface\left(t_{0}\right)},$$

where intracellular (t) is the fluorescence signal remaining after acid wash at the specified time and represents the endocytosed receptors; total(t) represents the fluorescence signal after PBS wash including cell surface and internalized signals; $surface\left(t_{0}\right)-total\left(t\right)$ represents the endocytosed or degraded receptor fluorescence signal inside cells.

In parallel experiments, 5H9 cells were serum-starved for 30 min, placed on ice, and incubated with AF488-conjugated transferrin (50 μg/ml) for 45 min. Cells were warmed to 37°C and incubated with NSLIT2 or CSLIT2 for different time periods. Endocytosis assays were performed as described above for ROBO1.

### Cell spreading assay

Human blood monocyte-derived macrophages, RAW264.7 cells, or COS-7 cells were grown to 90% confluence, serum-starved for 30 min, detached, and exposed to NSLIT2 or bio-inactive CSLIT2 or ΔD2SLIT2 for 15 min. ΔD2SLIT2 contains the N-terminal fragment of SLIT2 but lacks the D2 LRR domain that is essential for ROBO1 binding, and cannot bind to ROBO1 receptor (Patel et al, 2012; Bhosle et al, 2020). Cells (0.5−1 × 10^5^) were added to poly-D-lysine–precoated coverslips in 12-well plates for 1 h in serum-free RPMI-1640 medium and allowed to attach and spread. Cells were fixed with 4% PFA and permeabilized with 0.1% Triton X-100. Cells were incubated with AF488-phalloidin or AF488-phalloidin together with DAPI and mounted for immunofluorescence microscopy.

### Proximity ligation assay

COS-7 cells (3−5 × 10^4^) were grown on eight-well chamber glass slides (BD Falcon eight-chamber tissue culture-treated glass slides, Ref 354118; Corning) in serum-free RPMI-1640. PLAs were performed according to the manufacturer’s instructions. All incubations were performed in a black humidified chamber starting from the blocking step. Briefly, cells were incubated with NSLIT2 or CSLIT2 in serum-free medium for 15 min and samples were washed twice with PBS and fixed with 4% PFA. After washing three times with PBS, cells were permeabilized with 0.1% Triton X-100 in PBS for 15 min, washed again three times, and incubated with the blocking buffer provided by the kit for 1 h at 37°C. Slides were incubated with primary antibodies at 4°C overnight, washed three times with buffer A (0.01 M Tris, 0.15 M NaCl, 0.05% Tween-20, pH 7.4), and incubated with secondary antibodies at 37°C for 1 h. Slides were processed for the ligation for 30 min at 37°C, and washed twice with buffer A, and amplification was done for 100 min at 37°C. Slides were washed twice with buffer B (0.2 M Tris, 0.1 M NaCl, pH 7.5) and once with 0.01 x wash buffer B. Mounting medium with DAPI (ab104139; Abcam) was added to the slides and microscopy performed using a Quorum Spinning Disk Confocal microscope. Imaging data were analyzed by Volocity 6.3 software using the same criterion setting as in BlobFinder software (Allalou & Wählby, 2009). Interactions were quantified by counting the number of dots per nucleus, as well as the intensity of the signal per dot. An increase in intensity is the consequence of a concentration of interactions in the same cellular dots (Gauthier et al, 2015).

### Immunoblotting

Cells were lysed using ice-cold RIPA lysis buffer with protease inhibitor cocktail (Sigma-Aldrich). Samples were run on SDS–PAGE, transferred to 0.45-μm PVDF membrane (Millipore), and probed with specific primary antibodies and the corresponding HRP-conjugated goat anti-mouse or anti-rabbit secondary antibodies. The membrane was washed with TBST, treated with SuperSignal Chemiluminescent Substrate (Thermo Fisher Scientific), and visualized on a ChemiDoc MP imaging system (Bio-Rad Laboratories). Band intensity was quantified using ImageJ software, version 1.53k.

### Immunofluorescence microscopy imaging acquisition and analysis

Immunofluorescence microscopy was performed using a Quorum Spinning Disk Confocal microscope Leica DMi8 (Leica Microsystems) equipped with a Hamamatsu C9100-13 EMCCD camera and 40× (NA-1.3) oil immersion objectives for endocytosis and cell spreading assays. For PLAs, a 40× (NA-1.1) water immersion objective was used. For all experiments, 7–15 random fields for each experimental condition were imaged as Z-stacked images. Image analyses were performed using Volocity 6.3 software (PerkinElmer).

### BioID sample processing

BioID (Roux et al, 2012) was performed as described previously (Gingras et al, 2019; Astori et al, 2020). In brief, after selection (RPMI-1640 + 10% FBS + 200 μg/ml hygromycin B), two independent replicates of five 15-cm plates of subconfluent (60%) BirA*-FLAG or ROBO1-BirA*-FLAG fusion-expressing cells were incubated for 24 h in complete medium supplemented with 1 μg/ml tetracycline (Sigma-Aldrich) and 50 μM biotin (BioShop Canada). Cells were incubated with purified NSLIT2 (30 nM) for 24 h together with tetracycline. Cells were collected and pelleted (600*g*, 3 min). Pellets were washed twice with PBS, and dried pellets were snap-frozen. The cell pellets were resuspended in 10 ml of lysis buffer (50 mM Tris–HCl, pH 7.5, 150 mM NaCl, 1 mM EDTA, 1 mM EGTA, 1% Triton X-100, 0.1% SDS, 1:500 protease inhibitor cocktail, 1:1,000 Benzonase nuclease [Novagen, Sigma-Aldrich]) and incubated on an end-over-end rotator at 4°C for 1 h, briefly sonicated to disrupt any visible aggregates, then centrifuged at 45,000*g* for 30 min at 4°C. The supernatant was transferred to a fresh 15-ml conical tube. 30 μl of packed, pre-equilibrated streptavidin-Sepharose beads (GE) was added, and the mixture was incubated for 3 h at 4°C with end-over-end rotation. Beads were pelleted by centrifugation at 600*g* for 2 min and transferred with 1 ml of lysis buffer to a fresh Eppendorf tube. Beads were washed once with 1 ml lysis buffer and twice with 1 ml of 50 mM ammonium bicarbonate (pH = 8.3). Beads were transferred in ammonium bicarbonate to a fresh centrifuge tube and washed two more times with 1 ml ammonium bicarbonate buffer. Tryptic digestion was performed by incubating the beads with 1 μg MS-grade TPCK trypsin (Promega) dissolved in 200 μl of 50 mM ammonium bicarbonate (pH 8.3) overnight at 37°C. The next morning, 0.5 μg MS-grade TPCK trypsin was added, and beads were incubated for an additional two hours at 37°C. Beads were pelleted by centrifugation at 2,000*g* for 2 min, and the supernatant was transferred to a fresh Eppendorf tube. Beads were washed twice with 150 μl of 50 mM ammonium bicarbonate, and these washes were pooled with the first eluate. The sample was lyophilized and resuspended in buffer A (0.1% formic acid). 1/5th of the sample was analyzed per MS run.

### BioID LC-MS

Peptides were subjected to liquid chromatography performed on precolumns (150-mm I.D.) and analytical columns (75-mm I.D.) assembled in-house using fused silica capillary tubing (InnovaQuartz) packed with 100 Å C18 (Magic, Michrom Bioresources). A 120-min reversed-phase chromatographic gradient (0–100% CAN, 0.1% HCOOH) running at 250 nl/min on a Proxeon EASY-nLC pump was applied to samples in-line with a hybrid LTQ-Orbitrap Velos mass spectrometer (Thermo Fisher Scientific). A parent ion scan was performed in the Orbitrap at a 60,000 resolution (fwhm), with up to the 20 most intense precursors being selected for MS/MS (minimum ion count of 1,000 for activation), using standard collision-induced dissociation fragmentation. Dynamic exclusion was activated such that MS/MS of the same m/z (within a range of 15 ppm; exclusion list size = 500) detected twice within 15 s was excluded from analysis for 30 s. For protein identification, RAW files were converted to the. mzXML format using ProteoWizard (Holman et al, 2014), then searched using X!Tandem (Craig & Beavis, 2004) against the human database (Human RefSeq Version 45). Parent ion mass tolerance error of 15 ppm and fragment ion mass of 0.4 Da were indicated. No fixed modifications were included, but oxidation (M), acetylation (protein N terminus), and deamidation (N, Q) were included as potential modifications. Each sample was analyzed using two technical replicates. Data were analyzed using Trans-Proteomic Pipeline (Keller et al, 2005) via the ProHits software suite (Liu et al, 2010). Proteins identified with a ProteinProphet cutoff of 0.9 were analyzed with the SAINT algorithm (Choi et al, 2011) using 4 BirA*-FLAG samples as negative controls (12 BirA*-FLAG–only controls compressed to 4). High-confidence interactors were defined as those with Bayesian false discovery rate ≤0.01 (Table S3).

Table S3. ROBO1 BioID.

### Statistical analyses

Statistical analyses were performed using GraphPad Prism 8 software, and the results were presented as the mean ± standard deviation. The data fulfilled the requirements for normal distribution using the Shapiro-Wilk test. A two-tailed paired *t* test was used for two-group comparisons. One-way ANOVA followed by Tukey’s post hoc multiple comparison test was used for multiple-group comparisons with a single independent variable, and two-way ANOVA followed by Sidak’s post hoc multiple comparison test was used for multiple-group comparisons with two independent variables. A *P*-value of less than 0.05 was considered statistically significant.

## Supplementary Material

Reviewer comments

## Data Availability

Raw mass spectrometry data have been uploaded to the MassIVE public repository (https://massive.ucsd.edu/) under accession # MSV000092650.
