# Supplementary material for: APC regulates NSLIT2-induced internalization and signaling of the chemorepellent receptor, ROBO1
Source: Life Sci Alliance. 2026 Nov 10;10(1):e202403190. doi: 10.26508/lsa.202403190 (PMC13755696; doi:10.26508/lsa.202403190)
Supplement: Supplementary file 1 [file LSA-2024-03190_review_history.pdf]

APC regulates NSLIT2-induced internalization and signaling of the chemorepellent receptor, ROBO1

Yi-Wei Huang, Zhubing Li, Jonathan St-Germain, Bo Pang, Richard Collins, Etienne Coyaud, Wenjuan Li, Sajedabanu Patel, Nourhan Almasri, Amir Mohamed, Brian Raught, Sergio Grinstein, and Lisa Robinson
DOI: https://doi.org/10.26508/lsa.202403190

Corresponding author(s): Lisa Robinson, Hospital for Sick Children

Review Timeline:

Submission Date:	2024-12-24
Editorial Decision:	2024-12-26
Revision Received:	2026-07-18
Editorial Decision:	2026-08-10
Revision Received:	2026-10-14
Accepted:	2026-10-16

Scientific Editor: Tim Fessenden

Transaction Report:

(Note: With the exception of the correction of typographical or spelling errors that could be a source of ambiguity, letters and reports are not edited. The original formatting of letters and referee reports may not be reflected in this compilation.)

Please note that the manuscript was reviewed at *Review Commons* and these reports were taken into account in the decision-making process at *Life Science Alliance.*

Review COMMONS

Reviews

Review #1

Summary:

The authors investigate the endocytosis of the axon guidance receptor ROBO1 that is triggered by binding to its ligand SLIT2 and required for its ligand-induced inhibition of cell spreading. For this, they perform internalization experiments in a cell line expressing extra- and intracellularly tagged ROBO1. Using BioID they identify the protein APC as novel regulator of ROBO1 endocytosis. Their main finding is that SLIT2 induces the dissociation of APC thereby promoting endocytosis.

Major comments:

The data and key conclusions of the paper are convincing. However, the reliability of the findings in terms of the new interaction could be improved by not relying solely on proximity ligation approaches (BioID, PLA), but employing a complementary biochemical strategy.

The authors state that an immunoprecipitation (IP) was not possible due to a lack of antibodies for IP. This does not seem convincing since in the paper Saito-Diaz et al which they cite commercial antibodies were used to immunoprecipitate APC. Alternatively, the cell line expressing tagged ROBO1 could be used together with endogenous or tagged APC for an biochemical interaction experiment.

Regarding the PLA experiment, I was very surprised by the very strong labeling for Clathrin+ROBO1 shown in the representative image. It is hard to believe that this image is representative when the average number of dots in the quantification is about 100. From the image it is also hard to see how it would be possible to quantify individual dots. For this, a zoom would be helpful.

Clathrin and ROBO1 are likely not even direct interactors but come together by their common interaction with AP2. Therefore, to back this surprisingly strong result up, I would recommend to include one more control such as another rabbit antibody recognizing a protein that does not associate with clathrin or use e.g. the ROBO1 wildtype vs the ROBO1 mutant, that does not bind AP2 and therefore should also not associate with clathrin, for the experiment. Even better, the authors could confirm the PLA results by the mentioned complementary biochemical experiments to bolster the findings by an independent approach.

Minor comments:

In general, data and methods are presented in a manner that should make them reproducible by others. Some small things to improve are:

- In the paragraph on antibodies the used concentrations for the different applications should be provided.
- It should be described how the poly-D-lysine coating was exactly performed.

The statistical analysis looks adequate. There are just some minor things that should be specified:

- Just to make sure: Is it really always SD which is provided and not SEM? Sometimes the error bars look so small that I was wondering about this.
- It should be specified for each experiment which post-hoc test is used or stated that one is always used for the One-Way ANOVA and the other for the Two-Way ANOVA resp. a rationale should be provided why two different post-hoc tests are used.
- When using the t-test, it should be stated whether it is paired or unpaired and one- or two-tailed.
- It should be stated whether it was tested that the data fulfill the requirements for parametric tests (normal distribution).

Text and figures are mostly clear, apart from some small things:

- I was wondering about figure 1B. If I understand the methods description right, all cells were permeabilized prior to

secondary antibody application. Why then is so little fluorescence for Flag visible in the first PBS row at 30 min? That would only make sense for me if the cell was not permeabilized and the protein internalized. So where did the majority of the protein end up after 30 min since you should see the entire population in a permeabilized cell? Could you please comment on this?
- Fig. 2A the upper left image (0 min PBS) should be very similar to the upper left image in Fig. 1B, shouldn´t it? But it looks quite different to me in terms of surface amount of ROBO1-Flag. Could you please comment on this?
- Please explain what the molecular difference between bio-active NSLIT2 and bio-inactive CSLIT2 is. Please provide a rationale why you sometimes use CSLIT2 as negative control and sometimes ∆D2SLIT2. In Fig. 3G you are using ∆D2SLIT2. Even though there is no significance reached with the analyzed n, it is very striking that the bars are consistently higher upon ∆D2SLIT2 application. Can you comment on this effect? Or am I misunderstanding the labeling of the figure?
- On page 3 it states "...endocytosis of ROBO1...requires...APC": I found this confusing since it is the dissociation of APC that is required for promoting endocytosis. Therefore, it would be good to rephrase this sentence.
- On page 8 is written "...cells surface ROBO1 [is] removed". Please be more accurate since the acid wash does not remove ROBO1, but only the antibody bound to the extracellular epitope.
- On page 8 provide an explanation for the abbreviation HAC.
- On page 15 you speak of "mutant AP2". Please be more accurate since there is no mutant AP2 involved, but you are refering to ROBO1 with mutations in its AP2 binding motifs.
- On page 14 you speak of "cells expressing the mutant alleles of AP2". As above, please be more accurate and replace with "cells expressing ROBO1 harboring mutations in both AP2 binding sites".
- On page 19 you write: "Using proximity ligation assays, we observed that ROBO1, APC and clathrin interact with one another". I am maybe a bit picky here, but in my eyes with these assays you only show that they are very close together and might be in a complex, but you do not show (direct) interaction in a strict sense. Therefore, I would downtone this a bit.
- In Fig. 5B I would find it easier for the reader if siRNA and control were shown side by side for the different conditions.
- Between the internalization assays and the spreading assays, you switch from HEK293 cells to COS7 cells. Please provide a rationale for this for the reader.
- You provide a table with putative interactors within the paper and as supplementary table. Could you please explain better to the reader what your criteria were for including hits into the "short-list" presented in Table1.

Typos

- p. 6: CO2 instead of CO2
- p21 last line: Immunoblotting should not be capitzalized.
- Figure s1 legend: full-lenth is missing a g

Referees cross-commenting

I find the comments of Reviewer 2 productive and reasonable, and they overlap in part with mine (e.g. regarding biochemical approaches such as co-IP).

It was already known from Drosophila and for mammalian cells that SLIT2 induces the endocytosis of ROBO1 and that this is necessary for its repulsive function in axon guidance as the authors point out. The key advance of the study is the identification of APC as an interactor of ROBO1 which decreases its endocytosis until it dissociates upon SLIT2 binding to ROBO1. This is an interesting aspect which opens up parallels to the regulation of Wnt signaling by APC as the authors discuss. The significance of this finding would be even greater if it would have been shown that this mechanism actually operates in axon guidance. That not being the case, the authours might want to discuss in more detail if APC has previously been implicated to affect axon guidance.
Researchers working on endocytosis, adhesion, cellular signaling and the development of the nervous system will be interested in these findings.

Between 1 and 3 months
Yes

Review #2

Summary:

In this paper, the authors sought to identify the mechanism of Slit2-induced hRobo1 internalization and its signaling. They demonstrated that Slit2-induced hRobo1 internalization is regulated by one of the Robo1 C-terminal binding partners, adenomatous polyposis coli (APC). By using various in vitro experiments, the authors have concluded that APC constitutively interacts with hRobo1, and this interaction is disrupted upon the binding of Slit2 to the extracellular domain of hRobo1. They also showed that the dissociation of interaction between APC and hRobo1 is important for clathrin-mediated endocytosis of hRobo1 and subsequent cell morphology.
In conclusion, while this study presents intriguing findings, there are notable experimental concerns. In several instances, the authors fail to sufficiently elucidate the experimental setup or provide specific conditions for certain experiments, which may pose challenges for readers in understanding the methodology thoroughly. Also, the labels for the figures can be more accurate and clearly stated.

Major comments:

1. As the authors emphasize the role of NSlit2 in Robo1 internalization throughout their manuscript, I suggest authors include "NSlit" in their title. Something like this "Adenomatous polyposis coli (APC) regulates the NSlit2- induced internalization and signaling of the chemo repellent receptor, hRoundabout (ROBO) 1" or maybe a better title.
2. In addition to transferrin as the control for their internalization studies, have the authors tested the specificity of NSlit-2-induced internalization with other Robo receptors such as Robo2? Does the APC bind to Robo2 also?
3. The N-Slit group at 0' in Figure 1 b and Figure 2a, the Flag-Robo staining looks very different. Is it because the authors did not use ADAM protease inhibitor in Figure 2a that's why they are seeing more internalized Flag-Robo at 0'? It is not very clear either in the Results or the legend.
4. Have the authors tested the Surface Robo1 pool in siAPC cells induced with or without N-Slit2?
5. Does the Robo1 mutated with AP2 binding motifs interact with APC? Have authors performed a Proximity ligation assay with AP2-binding motifs mutated Robo1 and APC?
6. The resolution of PLA dots in the current version is very low. Authors should include higher magnification pictures for these interactions and also PLA dots channel should be separately represented in addition to the DAPI merged images for better clarity and interpretation.
7. Do the Slit2 treated cells affect APC mRNA expression? Or does Slit2 only inhibit the interaction between APC and Robo1? Have the authors tested the mRNA expression of APC in slit2-treated and untreated cells?
8. The authors have tested the effect of Slit2-induced inhibition of cell spreading under different experimental conditions however it is also important to test the cell migration/proliferation rates under control and siAPC conditions with or without Slit2 treatment.
9. Do authors see the inhibition of Robo1 and Cyfip interactions also in the presence of Slit2 by PLA assay?
10. Studying the endogenous Robo1 and APC interaction by PLA is good but I suggest authors do standard co-IP assays to visualize these interactions since authors have already generated a variety of general epitope- tagged constructs for both Robo1 and APC. These epitope-specific antibodies that are best suitable for IP are easily available with many antibody companies. This is the first study to suggest that the interaction between Robo1 and APC so the strong biochemistry would have a good impact on the findings.

Minor comments:

1. I suggest the authors show the single-channel images of Flag-robo (green) in Figure 2B for a clear visualization of internalized Robo in a cell. With DAPI-merged images, it is hard to specifically visualize Robo in these cells.
2. In Figure 1C, the Y axis should have a clear indication. Instead of "% internalized" it should be mentioned as "% Internalized Robo1".
3. I suggest authors to include the simple schematic of the mechanism they are proposing in the manuscript.
4. The authors should mention the rationale or the function of using the acid wash method for their experimental conditions for a better understanding of the reader.
5. siRNA-mediated knockdown of specific genes should be correctly denoted in the figure. For example, instead of "CLTC", it should be "siCLTC" for easy understanding. The same correction has to be done in all the figures with siRNA data.

Referees cross-commenting

Reviewer 1 comments and suggestions are valid and carry significant weight in improving the manuscript.

Strengths: The manuscript writing is good and the authors have generated a lot of constructs for a thorough understanding of Robo1 internalization events under different conditions. Studying the differential protein interactions with and without Slit2 with the Robo1-Bir*Flag method is convincing.

Limitations: The representation of figures and their labels, Figure resolution, poor quality, missing important controls and experiments.

Advance: Not very conceptual

Audience: Broad and basic research

My field of expertise: Endocytosis, receptor surface labeling studies, ligand mediated receptor signaling and its effect on axon guidance during embryonic development.

Between 1 and 3 months
Yes

Review #3

In their manuscript, Robinson and colleagues explore the role of the APC protein in the regulation of Slit-Robo signaling in mammalian cell culture. In particular, the authors investigate the importance of APC in controlling the endocytosis of Robo and its subsequent signaling. The authors begin by demonstrating that as previously reported in a Drosophila model that Slit induces the internalization of Robo through Clathrin dependent endocytosis and that this effect depends on two AP binding motifs in the Robo c-terminal tail. In a cellular readout for Slit- signaling the authors demonstrate that Slit inhibition of cell spreading in COS7 cells depends on the receptor endocytosis. A BioID screen for proteins that bind Robo in a slit-dependent manner identified a number of candidate proteins and the authors chose to focus on the APC protein whose association with Robo is down-regulated by Slit. Using a series of in vitro and cell based experiments the authors propose a model in which APC negatively regulates Robo internalization and subsequently impacts receptor signaling. Overall the findings are interesting and many of the experiments are well controlled; however, significant technical and conceptual concerns limit enthusiasm for the manuscript in its current form.

General- throughout the manuscript the magnification of the cells in the micrographs are too low. I would recommend including insets to more clearly show the observed effects (especially for the localization figure).

Specific comments

Figure 1: The effect of Slit on Robo internalization appears to be robust and the transferrin control is welcome. The authors need to do a better job of explaining how exactly they are quantifying the internalized pool, especially given the likely cell to cell variability in the amount of expression of the transfected constructs. Labeling the figure more clearly to indicate what is Scarlet tagged would help and the purpose of the acid-washing procedure should be more clearly explained.

Figure 2: There seems to be a mismatch in the representative images for the Dyn treatments- both concentrations appear to reduce internalization of Robo R ~ 2 fold, but the images show essentially 0 internalized receptor. (2A and B)

Figure 3: The analysis of the Robo c-terminal AP binding motifs is a bit confusing. The authors refer to these manipulations as Robo alleles; however, my understanding is that these are over-expressed constructs in stably transfected lines. They are not 'alleles.' It is puzzling how the effects of Slit appear to be restricted to the transfected cells, especially given that all of these cells should be expressing endogenous Robo. Some clarification would be welcome.

Figure 4: This figure presents the 'validation' of the Robo APC interaction. There are major problems here. First, PLA is not an adequate substitute for biochemical interaction and it does not allow for clear documentation of ligand-dependent effects. To suggest as the authors do that this analysis provides evidence for a multi-protein complex between Robo, APC and clathrin is not legitimate. Furthermore, the nature of the PLA assay and what they are counting is unlear and misleading. In the methods the author's state

'Interactions were quantified by counting the number of dots per nucleus as well as the intensity of the signal per dot. An increase in intensity is the consequence of a concentration of interactions in the same cellular dots (Gauthier et al., 2015)'

Surely, we are not to believe that nuclear PLA signals would exist between this transmembrane protein and endocytic machinery.

The authors should perform co-IP experiments +/- Slit to validate their findings from BioID.

Figures 5 and 6: These data represent the functional analysis of APC's role in Robo signaling. There are several observations that do not it with the model which states that APC associates with Robo to prevent endocytosis until Slit arrives. For example, this model would predict that loss of APC should lead to an increase in Robo internalization and an increase in Robo dependent inhibition of cell spreading- neither of these predictions match their data.

In their manuscript, Robinson and colleagues explore the role of the APC protein in the regulation of Slit-Robo signaling in mammalian cell culture. In particular, the authors investigate the importance of APC in controlling the endocytosis of Robo and its subsequent signaling. The authors begin by demonstrating that as previously reported in a Drosophila model that Slit induces the internalization of Robo through Clathrin dependent endocytosis and that this effect depends on two AP binding motifs in the Robo c-terminal tail. In a cellular readout for Slit- signaling the authors demonstrate that Slit inhibition of cell spreading in COS7 cells depends on the receptor endocytosis. A BioID screen for proteins that bind Robo in a slit-dependent manner identified a number of candidate proteins and the authors chose to focus on the APC protein whose association with Robo is down-regulated by Slit. Using a series of in vitro and cell based experiments the authors propose a model in which APC negatively regulates Robo internalization and subsequently impacts receptor signaling. Overall the findings are interesting and many of the experiments are well controlled; however, significant technical and conceptual concerns limit enthusiasm for the manuscript in its current form.

Cannot tell / Not applicable

No

December 26, 2024

Re: Life Science Alliance manuscript #LSA-2024-03190

Dr. Lisa A. Robinson
The Hospital for Sick Children
Program in Cell Biology
555 University Avenue, Room 5265 The Hospital for Sick Children
Toronto, Ontario M5G 1X8
Canada

Dear Dr. Robinson,

Thank you for submitting your manuscript entitled "Adenomatous polyposis coli (APC) regulates the NSLIT2-induced internalization and signaling of the chemorepellent receptor, Roundabout (ROBO) 1" to Life Science Alliance. We invite you to submit a revised manuscript, according to your Revision Plan.

To upload the revised version of your manuscript, please log in to your account: https://lsa.msubmit.net/cgi-bin/main.plex

You will be guided to complete the submission of your revised manuscript and to fill in all necessary information. Please get in touch in case you do not know or remember your login name.

While you are revising your manuscript, please also attend to the below editorial points to help expedite the publication of your manuscript. Please direct any editorial questions to the journal office.

The typical timeframe for revisions is three months. Please note that papers are generally considered through only one revision cycle, so strong support from the referees on the revised version is needed for acceptance.

When submitting the revision, please include a letter addressing the reviewers' comments point by point.

Thank you for this interesting contribution to Life Science Alliance. We are looking forward to receiving your revised manuscript.

Sincerely,

Eric Sawey, PhD
Executive Editor
Life Science Alliance
http://www.lsajournal.org

A. THESE ITEMS ARE REQUIRED FOR REVISIONS

-- A letter addressing the reviewers' comments point by point.

-- An editable version of the final text (.DOC or .DOCX) is needed for copyediting (no PDFs).

-- High-resolution figure, supplementary figure and video files uploaded as individual files: See our detailed guidelines for preparing your production-ready images, https://www.life-science-alliance.org/authors

-- Summary blurb (enter in submission system): A short text summarizing in a single sentence the study (max. 200 characters including spaces). This text is used in conjunction with the titles of papers, hence should be informative and complementary to the title and running title. It should describe the context and significance of the findings for a general readership; it should be written in the present tense and refer to the work in the third person. Author names should not be mentioned.

-- By submitting a revision, you attest that you are aware of our payment policies found here: https://www.life-science-alliance.org/copyright-license-fee

B. MANUSCRIPT ORGANIZATION AND FORMATTING:

Full guidelines are available on our Instructions for Authors page, https://www.life-science-alliance.org/authors

We encourage our authors to provide original source data, particularly uncropped/-processed electrophoretic blots and spreadsheets for the main figures of the manuscript. If you would like to add source data, we would welcome one PDF/Excel-file per figure for this information. These files will be linked online as supplementary "Source Data" files.

IMPORTANT: It is Life Science Alliance policy that if requested, original data images must be made available. Failure to provide original images upon request will result in unavoidable delays in publication. Please ensure that you have access to all original microscopy and blot data images before submitting your revision.

Point-by point response to reviewers

Manuscript number: LSA-2024-03190
Corresponding author(s): Lisa A. Robinson

We sincerely thank the reviewers for their comprehensive and constructive feedback. Below, we submit our point-by-point response (in bold) to the reviewers' comments and revisions addressed in this submission as per the revision plan submitted.

Reviewer #1

Major comments:

1) The data and key conclusions of the paper are convincing. However, the reliability of the findings in terms of the new interaction could be improved by not relying solely on proximity ligation approaches (BioID, PLA), but employing a complementary biochemical strategy. The authors state that an immunoprecipitation (IP) was not possible due to a lack of antibodies for IP. This does not seem convincing since in the paper Saito-Diaz et al which they cite commercial antibodies were used to immunoprecipitate APC. Alternatively, the cell line expressing tagged ROBO1 could be used together with endogenous or tagged APC for an biochemical interaction experiment.

   We thank the reviewer for this important suggestion. In our initial studies, we attempted co-immunoprecipitation (co-IP) experiments using several different antibodies directed to APC. The signal detected was very low, possibly reflecting relatively low endogenous expression of ROBO1 in COS-7 cells, technical challenges associated with co-IP of APC and ROBO1, which are both large proteins (>200 kDa), and/or transient interactions between the two proteins.

   As per the revision plan, we peformed co-IP experiments using HEK293A cells stably expressing full length ROBO1 (5H9 cells). We note that the APC antibody used for immunoprecipitation in the study by Saito-Diaz et al. (Santa Cruz, sc-7930) has since been discontinued and was therefore not available for our studies. Consequently, we explored several alternative approaches using commercially available APC antibodies: Mouse anti-APC (Abcam- ab16794), Rabbit anti-APC (Abcam- ab40778), Mouse anti-APC (EMD, cat# OP44) and Rabbit anti-APC (Invitrogen, cat # PA5-30580). Despite testing these reagents under multiple conditions, we were unable to detect a convincing APC signal suitable for co-immunoprecipitation analysis. These findings highlight the technical challenges associated with biochemical detection of APC-containing complexes and prevented us from obtaining a reliable co-IP result to complement our proximity-based interaction studies.

2) Clathrin and ROBO1 are likely not even direct interactors but come together by their common interaction with AP2. Therefore, to back this surprisingly strong result up, I would recommend to include one more control such as another rabbit antibody recognizing a protein that does

not associate with clathrin or use e.g. the ROBO1 wildtype vs the ROBO1 mutant, that does not bind AP2 and therefore should also not associate with clathrin, for the experiment. Even better, the authors could confirm the PLA results by the mentioned complementary biochemical experiments to bolster the findings by an independent approach.

We thank the reviewer for this valuable suggestion. As noted in our response to comment (1), despite extensive efforts, we were unable to obtain reliable co-immunoprecipitation data to independently validate the association between ROBO1 and clathrin. Therefore, we pursued alternative approaches, performing additional proximity ligation assay (PLA) experiments to further assess the specificity of this interaction.

First, we performed PLA using antibodies directed to ROBO1 and clathrin in HEK293A cells expressing full-length ROBO1 (5H9 cells) and detected a robust positive signal, supporting the spatial proximity between these proteins. These data have been included in Figures s4A and s4B and are described on page 17 lines 448-449 of the revised manuscript.

To address the reviewer's suggestion for a negative control, we assessed the proximity between clathrin and p38 MAPK, a protein not known to associate with the clathrin assembly machinery. This antibody pair did not produce a detectable PLA signal, supporting the specificity of the ROBO1–clathrin PLA results. These data are also presented in Figures s4C and s4D and are described on page 17 lines 449-454 of the revised manuscript. Taken together, these additional experiments provide further support for a specific association between ROBO1 and clathrin.

3) Regarding the PLA experiment, I was very surprised by the very strong labeling for Clathrin+ROBO1 shown in the representative image. It is hard to believe that this image is representative when the average number of dots in the quantification is about 100. From the image it is also hard to see how it would be possible to quantify individual dots. For this, a zoom would be helpful.

We thank the reviewer for this helpful comment. In the revised manuscript, we have added a magnified panel in Figure 4E.

Significance

It was already known from Drosophila and for mammalian cells that SLIT2 induces the endocytosis of ROBO1 and that this is necessary for its repulsive function in axon guidance as the authors point out. The key advance of the study is the identification of APC as an interactor of ROBO1 which decreases its endocytosis until it dissociates upon SLIT2 binding to ROBO1. This is an interesting aspect which opens up parallels to the regulation of Wnt signaling by APC as the authors discuss. The significance of this finding would be even greater if it would have been shown that this mechanism actually operates in axon guidance. That not being the case, the authours might want to discuss in more detail if APC has previously been implicated to affect axon guidance.

Researchers working on endocytosis, adhesion, cellular signaling and the development of the nervous system will be interested in these findings.

We thank the reviewer for the positive comments regarding the significance of our findings. As recommended, the Discussion section of the revised manuscript has been modified to discuss the role of APC in axon guidance. Please see page 20 lines 535-540.

Reviewer #1

Minor comments:

1) In the paragraph on antibodies the used concentrations for the different applications should be provided.

   We thank the reviewer for this suggestion and apologize for the omission. In the revised manuscript, we have added a supplementary table to clarify the concentrations of antibodies used for different experimental applications. Please see Table s1.

2) It should be described how the poly-D-lysine coating was exactly performed.

   We thank the reviewer for this comment. Glass coverslips were pre-coated with poly-D-lysine (50 µg/mL; MW 70-150 kDa, P6407, Sigma-Aldrich) for 30 min at 37°C prior to using them for experiments. In the revised manuscript, we have added the procedure for poly-D-lysine coating in the "Materials and Methods" section. Please see page 8 line 190-191.

3) The statistical analysis looks adequate. There are just some minor things that should be specified:

   i. Just to make sure: Is it really always SD which is provided and not SEM? Sometimes the error bars look so small that I was wondering about this.

      We appreciate the opportunity to clarify that we used SD consistently in the manuscript. We have specificed this on page 12, line 321 of the "Materials and Methods" section.

   ii. It should be specified for each experiment which post-hoc test is used or stated that one is always used for the One-Way ANOVA and the other for the Two-Way ANOVA resp. a rationale should be provided why two different post-hoc tests are used.

      We have added the post hoc tests used for each experiment in the corresponding figure legend. The rationale for the different post hoc tests used has also been added in the "Materials and Methods" section as "Two-tailed paired Student's *t*-test was used for two-group comparisons. One-way ANOVA followed by Tukey's post hoc multiple comparison test was used for multiple-group comparisons with a single independent variable, and two-way ANOVA followed by Sidak's post hoc multiple comparison test was used for multiple-group comparisons with two

independent variables". Please see page 12 line 323-326, page 22 line 607-608, 612-613, page 23 lines 621-622, 625-626, 628-629, 631-632, 639-640, 643-644, page 24 lines 649-650, 664-665, 669-670, 672-673, page 25 lines 677-678, 682-684, 687-688, page 26 lines 713-714, 718-719, 724-725, 727-728, page 27, lines 735, 739-741, 744-746, 750-751.

iii. When using the t-test, it should be stated whether it is paired or unpaired and one- or two-tailed.

Two-tailed paired Student's *t*-test was used in Fig. 5C. We have added this clarification to the "Materials and Methods" section and figure legend in the revised manuscript. Please see page 12 line 322-323, page 24 line 675-676.

iv. It should be stated whether it was tested that the data fulfill the requirements for parametric tests (normal distribution).

We thank the reviewer for this suggestion. We have now added "The data fulfilled the requirements for normal distribution using the Shapiro-Wilk test" in the "Materials and Methods" section in the revised manuscript. Please see page 12 line 321-322 in the revised manuscript.

4) Text and figures are mostly clear, apart from some small things:

i. I was wondering about figure 1B. If I understand the methods description right, all cells were permeabilized prior to secondary antibody application. Why then is so little fluorescence for Flag visible in the first PBS row at 30 min? That would only make sense for me if the cell was not permeabilized and the protein internalized. So where did the majority of the protein end up after 30 min since you should see the entire population in a permeabilized cell? Could you please comment on this?

We thank the reviewer for this important comment. The cells were permeabilized prior to secondary antibody application. Since NSLIT2 binding to ROBO1 can facilitate ADAM10-mediated ROBO1 cleavage to release the extracellular domain of ROBO1 (Coleman et al., 2010), this may have diminished visible signal for FLAG in the first PBS row at 30 min. In the revised manuscript we have added a comment about the finding described. Please see page 13 lines 341-343.

ii. Fig. 2A the upper left image (0 min PBS) should be very similar to the upper left image in Fig. 1B, shouldn´t it? But it looks quite different to me in terms of surface amount of ROBO1-Flag. Could you please comment on this?

We apologize for the confusing images included in the original version of the manuscript. As noted, the upper left image (0 min PBS) in Fig. 2A should be very similar to the upper left image in Fig. 1B. In the revised manuscript we have now

included an image for Fig. 2A that is more representative of the data from all the experiments we performed.

iii. Please explain what the molecular difference between bio-active NSLIT2 and bio-inactive CSLIT2 is. Please provide a rationale why you sometimes use CSLIT2 as negative control and sometimes ∆D2SLIT2. In Fig. 3G you are using ∆D2SLIT2. Even though there is no significance reached with the analyzed n, it is very striking that the bars are consistently higher upon ∆D2SLIT2 application. Can you comment on this effect? Or am I misunderstanding the labeling of the figure?

Bio-active NSLIT2 consists of the N-terminal fragment of SLIT2 and contains the second leucine-rich repeat (LRR) domain (D2), which binds to the first two Ig domains of the ROBO1 receptor (Ig1-2). Bio-inactive CSLIT2 consists of the C-terminal fragment of SLIT2, which does not bind ROBO1. ΔD2SLIT2 consists of the N-terminal fragment of SLIT2 but lacks D2 LRR domain that is essential for ROBO1 binding. Neither CSLIT2 nor ΔD2SLIT2 binds the ROBO1 receptor (Bhosle et al., 2020; Mukovozov et al., 2015; Patel et al., 2012).

We used CSLIT2 and ΔD2SLIT2 as negative controls in different experiments to validate the specificity of our findings using two independent approaches. CSLIT2 represents a naturally occurring C-terminal SLIT2 fragment that lacks the ROBO1-binding region, whereas ΔD2SLIT2 retains the N-terminal fragment but specifically lacks the D2 domain required for ROBO1 binding.

In Figure 3A and B, the surface area of cells exposed to CSLIT2 and ΔD2SLIT2 was very similar, and both were comparable to the surface area of cells exposed to control vehicle. In Figure 3F and G, the surface area of cells expressing wild-type ROBO1 was significantly reduced in the presence of NSLIT2 as compared to ΔD2SLIT2 since binding of NSLIT2 to ROBO1 induces cell rounding and inhibits cell spreading. Assays in Figures 3A–D were performed using COS-7 cells under basal conditions, whereas the spreading assays in Figures 3E-G were performed 24h after transfection with plasmids encoding wild-type ROBO1 or ROBO1 constructs containing mutations in the AP2-binding regions.

Importantly, as shown in Figures 3F and 3G, NSLIT2-induced inhibition of cell spreading required the presence of AP2-binding domains of ROBO1, whereas incubation with ΔD2SLIT2 did not significantly inhibit cell spreading irrespective of the presence or absence of AP2-binding domains of ROBO1. These data support the conclusion that the response requires the D2 domain and is dependent on ROBO1 binding. To avoid confusion, we have modified the X-axis labeling in Figures 3F and 3G to clarify that the spreading assays were performed following transfection of COS-7 cells with the indicated plasmids.

iv. On page 3 it states "...endocytosis of ROBO1...requires...APC": I found this confusing since it is the dissociation of APC that is required for promoting endocytosis. Therefore, it would be good to rephrase this sentence.

We apologize for the confusing language. In the revised manuscript, we have changed "endocytosis of ROBO1 from the cell surface requires the tumor suppressor protein, APC" to "endocytosis of ROBO1 from the cell surface requires the dissociation of the tumor suppressor protein, APC". Please see page 4 line 80-81 in the revised manuscript.

v. On page 8 is written "...cells surface ROBO1 [is] removed". Please be more accurate since the acid wash does not remove ROBO1, but only the antibody bound to the extracellular epitope.

We apologize for the confusing language. In the revised manuscript, we have changed "cell surface ROBO1 removed" to "anti-FLAG antibody binding ROBO1 removed from the cell surface". Please see page 8 line 200-202 in the revised manuscript.

vi. On page 8 provide an explanation for the abbreviation HAC.

To enhance clarity, in the revised manuscript we have used the full name "acetic acid" instead of using the abbreviation "HAC". Please see page 8 line 202 in the revised manuscript.

vii. On page 15 you speak of "mutant AP2". Please be more accurate since there is no mutant AP2 involved, but you are refering to ROBO1 with mutations in its AP2 binding motifs.
On page 14 you speak of "cells expressing the mutant alleles of AP2". As above, please be more accurate and replace with "cells expressing ROBO1 harboring mutations in both AP2 binding sites".

We thank the reviewer for noting this important point and apologize for the confusing language. For the sake of accuracy, we have made the changes suggested by the reviewer. Please see page 14 lines 376,379 and page 15 line 397-400 in the revised manuscript.

viii. On page 19 you write: "Using proximity ligation assays, we observed that ROBO1, APC and clathrin interact with one another". I am maybe a bit picky here, but in my eyes with these assays you only show that they are very close together and might be in a complex, but you do not show (direct) interaction in a strict sense. Therefore, I would downtone this a bit.

We thank the reviewer for this important comment. As suggested, in the revised manuscript, we replaced "Using proximity ligation assays, we observed that ROBO1, APC and clathrin interact with one another" with "Using proximity ligation assays, we observed that ROBO1, APC and clathrin are in close proximity to one another". Please see page 20 line 544. We have similarly amended the

language throughout the manuscript. Please see page 3 line 54-55, page 4 line 81, page 16 line 438, 441-442, 443-444, page 24 line 652 in the revised manuscript.

ix. In Fig. 5B I would find it easier for the reader if siRNA and control were shown side by side for the different conditions.

In the revised manuscript, we have made the changes suggested by the reviewer to enhance clarity. Please see revised Fig. 5B.

x. Between the internalization assays and the spreading assays, you switch from HEK293 cells to COS7 cells. Please provide a rationale for this for the reader.

Because the endogenous expression of ROBO1 is relatively low in COS-7 cells, we generated a HEK293A cell line that stably expresses ROBO1 and used these cells to examine subcellular traffic of ROBO1 and explore interactors of ROBO1. We next sought to explore the functional consequences of internalization of ROBO1 and the functional role of APC. As we and others previously showed that SLIT2-ROBO1 signaling inhibits cell spreading (Bhosle et al., 2020; Patel et al., 2012; Tole et al., 2009), we elected to use this measure as a biologic read-out. Because HEK293A cells do not spread as much as COS-7 cells, we instead used COS-7 cells for the spreading assays. We have included this information on page 6, lines 142-144

xi. You provide a table with putative interactors within the paper and as supplementary table. Could you please explain better to the reader what your criteria were for including hits into the "short-list" presented in Table1.

We thank the reviewer for this helpful suggestion. In the studies we performed, we chose proteins based on two criteria. The first was association with full-length ROBO1, but not with ROBO1 lacking the intracellular domain. The second was association with full-length ROBO1 under basal conditions, but loss of association with full-length ROBO1 after exposure of cells to NSLIT2. In the revised manuscript, we have added the criteria used to include 'hits' in the short-list. Please see page 15 line 410-413.

Typos
- p6: CO_2 instead of CO2
- p21 last line: Immunoblotting should not be capitzalized.
- Figure s1 legend: full-lenth is missing a g

We apologize for the oversight. In the revised manuscript, we have corrected these typos. Please see page 6 line 142, page 23 line 624 and page 25 line 700-701.

Reviewer #2

Major comments:

1) In addition to transferrin as the control for their internalization studies, have the authors tested the specificity of NSlit-2-induced internalization with other Robo receptors such as Robo2? Does the APC bind to Robo2 also?

   We thank the reviewer for this comment. Due to significant cost constraints, we focused our BioID experiments on identifying proteins that interact with ROBO1. We have modified the Discussion section of the revised manuscript to address this point and to highlight it as a future line of investigation. Please see page 22 lines 590-593 in the revised manuscript.

2) Does the Robo1 mutated with AP2 binding motifs interact with APC? Have authors performed a Proximity ligation assay with AP2-binding motifs mutated Robo1 and APC?

   We thank the reviewer for this valuable suggestion. As recommended, we performed proximity ligation assays (PLA) to investigate whether ROBO1 containing mutations in the AP2-binding motifs interacts with APC.

   To specifically detect the exogenously expressed receptors, we initially used anti-FLAG antibody (Sigma-Aldrich, F1804) in combination with anti-APC antibodies in cells stably expressing FLAG-tagged wild-type ROBO1 or ROBO1 carrying AP2-binding motif mutations (see Figure S1 for construct details). However, we were unable to detect a positive PLA signal under these conditions. We also tested anti-Myc antibody (Cell Signaling Technologies, 2276S) to detect the tagged ROBO1 constructs, but these experiments similarly failed to yield a relaible PLA signal. The absence of a PLA signal may reflect technical limitations rather than the absence of proximity between ROBO1 and APC. PLA requires that the epitopes recognized by the two antibodies are within approximately 40 nm of each other. Because the FLAG and Myc tags are located at the N-terminus of ROBO1, whereas APC is a cytoplasmic protein, the distance between the tagged epitope and APC may exceed the detection range of the assay. Furthermore, the three-dimensional conformation and membrane topology of ROBO1 may spatially separate the tagged extracellular epitopes from APC-associated intracellular domains, preventing sufficient proximity of the PLA probes even if ROBO1 and APC are located in close spatial proximity. In addition, epitope accessibility, fixation conditions, or antibody performance in PLA may further reduce signal detection.

   As an alternative approach, we performed PLA using antibodies directed to ROBO1 (MAB7118, R&D Systems) and APC (ab40778, Abcam). This combination allowed detection of a PLA signal in parental 5H9 cells expressing wild-type ROBO1, and in cells stably expressing ROBO1 with mutated AP2-binding motifs. However, because these antibodies also recognize endogenous ROBO1 and APC, the observed PLA signal likely reflects a combination of both endogenous and exogenous protein interactions. Therefore, while these results further support the proximity between ROBO1 and APC,

consistent with our observations in COS-7 cells (Figure 4), they do not allow us to conclusively determine whether mutation of the AP2-binding motifs affects the interaction between ROBO1 and APC. These additional data have been included in Figures s4E and s4F and are described in the revised manuscript page 17, lines 456-464.

To gain further insight, using antibodies directed to ROBO1 and clathrin, we performed PLA to investigate interactions between ROBO1 and clathrin in cells stably expressing either wild-type ROBO1 or ROBO1 carrying point mutations in the AP2-binding motifs. In cells expressing the AP2-binding mutant of ROBO1, there was a trend towards lower PLA signal, although the reduction was not statistically significant. These data have been included in Figure s4C and s4D of the revised manuscript. As noted above, this could reflect interaction between ROBO1 and clathrin via endogenous AP2 present in the cells expressing the AP2-binding mutant. It is also possible that interactions between ROBO1 and clathrin are not solely mediated by AP2. We have included discussion of these important considerations in the revised version of the manuscript. Please see page 17, lines 464-468.

3) Do the Slit2 treated cells affect APC mRNA expression? Or does Slit2 only inhibit the interaction between APC and Robo1? Have the authors tested the mRNA expression of APC in slit2-treated and untreated cells?

We thank the reviewer for this insightful question. To determine whether NSLIT2 regulates *APC* expression at the transcriptional level, we performed qPCR analysis of *APC* mRNA following exposure of cells to NSLIT2 for 4 h and 24 h. No significant changes in *APC* mRNA expression were observed at either time point, indicating that NSLIT2 does not regulate *APC* expression at the transcriptional level at the time points studied. Although very transient changes in *APC* mRNA cannot be ruled out, the lack of detectable differences at both 4 h and 24 h indicates that NSLIT2 does not measurably regulate *APC* transcription over these early and later time scales. These new data have been incorporated into the revised manuscript in Figure s5a and page 18, line 494-502.

Our findings are consistent with previous studies demonstrating that SLIT2–ROBO signaling primarily mediates rapid post-translational regulation of protein interactions and cytoskeletal dynamics rather than transcriptional regulation (Bhosle et al., 2020; Blockus and Chédotal, 2016).

4) Do authors see the inhibition of Robo1 and Cyfip interactions also in the presence of Slit2 by PLA assay?

We thank the reviewer for this interesting question. As this was beyond the scope of the current study, we did not examine whether NSLIT2 inhibits interactions between ROBO1 and CYFIP. In the Discussion section of the revised manuscript, this point has been addressed as a future line of investigation. Please see page 20 lines 540-542.

5) Studying the endogenous Robo1 and APC interaction by PLA is good but I suggest authors do standard co-IP assays to visualize these interactions since authors have already generated a variety of general epitope- tagged constructs for both Robo1 and APC. These epitope-specific antibodies that are best suitable for IP are easily available with many antibody companies. This is the first study to suggest that the interaction between Robo1 and APC so the strong biochemistry would have a good impact on the findings.

   We appreciate this important suggestion. Please also see our response to Reviewer 1, Major Comment 1. As noted there, we have tested multiple APC antibodies which have demonstrated weak or non-specific band signals in our studies. This could possibly have been due to the transient nature of the interaction, and hence the technical limitations prevented us from completing the co-immunoprecipitation assays. As an alternative approach, we performed the experiments described in the response to Reviewer 1, Comment 2, and Reviewer 2, Major Comment 2 above.

6) As the authors emphasize the role of NSlit2 in Robo1 internalization throughout their manuscript, I suggest authors include "NSlit" in their title. Something like this "Adenomatous polyposis coli (APC) regulates the NSlit2-induced internalization and signaling of the chemo repellent receptor, hRoundabout (ROBO) 1" or maybe a better title.

   As suggested, we have changed the title of the revised manuscript as to "APC regulates NSLIT2-induced internalization and signaling of the chemorepellent receptor, ROBO 1".

7) The N-Slit group at 0' in Figure 1 b and Figure 2a, the Flag-Robo staining looks very different. Is it because the authors did not use ADAM protease inhibitor in Figure 2a that's why they are seeing more internalized Flag-Robo at 0'? It is not very clear either in the Results or the legend.

   We apologize for the confusing images. We used ADAM protease inhibitor for all endocytosis assays, as mentioned in the "Materials and Methods" section. The upper left image (0 min PBS) in Fig. 2A should be very similar to the upper left image in Fig. 1B. We have now replaced the image in 2A with one that is more representative of the overall results. Please see our response to Reviewer 1, comment 4ii.

8) Have the authors tested the Surface Robo1 pool in siAPC cells induced with or without N-Slit2?

   We thank the reviewer for this important question. NSLIT2 was added to cells at the start of the endocytosis assay. At the time point of 0 min, the surface ROBO1 pool was not affected by NSLIT2. In the revised manuscript, we have now clarified this important point. Please see page 8 lines 196-198.

9) The resolution of PLA dots in the current version is very low. Authors should include higher magnification pictures for these interactions and also PLA dots channel should be separately represented in addition to the DAPI merged images for better clarity and interpretation.

   We thank the reviewer for these suggestions. In the revised manuscript, we have included figures with the recommended modifications to enhance clarity. Please see revised figure 4A, 4C, and 4E.

10) The authors have tested the effect of Slit2-induced inhibition of cell spreading under different experimental conditions however it is also important to test the cell migration/proliferation rates under control and siAPC conditions with or without Slit2 treatment.

   We thank the reviewer for this comment. In order to test the effect of APC on SLIT2-induced cell migration, a migratory cell type would be required. This would involve introducing a third cell type in addition to the HEK293 and COS-7 cells we have already used and additionally validating our key experimental findings in the new cell type. Please see our response to the 10th sub-comment in Minor Comment 4) of Reviewer 1. In the revised version of the manuscript, we have added this suggestion as an important future line of investigation. Please see page 21, lines 583-589 in the revised manscript.

Reviewer #2

Minor comments:

1) I suggest the authors show the single-channel images of Flag-robo (green) in Figure 2B for a clear visualization of internalized Robo in a cell. With DAPI-merged images, it is hard to specifically visualize Robo in these cells.

   We appreciate the reviewer's suggestion and assume they are referring to Figure 2A instead of 2B. To enhance clarity, in the revised manuscript, we have made the changes suggested by the reviewer.

2) In Figure 1C, the Y axis should have a clear indication. Instead of "% internalized" it should be mentioned as "% Internalized Robo1".

   We thank the reviewer for this suggestion and apologize for the oversight. In the revised manuscript, we have made the suggested change in Figure 1C, 2B, 2D, 2E, 5B, and 5D.

3) The authors should mention the rationale or the function of using the acid wash method for their experimental conditions for a better understanding of the reader.

   We thank the reviewer for this suggestion and apologize for the oversight. We performed acid wash experiments to remove the anti-FLAG antibody that binds ROBO1 from the cell surface for the endocytosis assay. To increase the clarity, in the "Materials and

Methods" section of the revised manuscript we have included the rationale for using acid wash. Please see page 8 line 200-202.

4) siRNA-mediated knockdown of specific genes should be correctly denoted in the figure. For example, instead of "CLTC", it should be "siCLTC" for easy understanding. The same correction has to be done in all the figures with siRNA data.

   We thank the reviewer for this helpful comment and apologize for the oversight. As suggested, we have made the suggested changes throughout the revised manuscript and in Figure 2C, 2D, 5A, 5B, 6A, 6B, 6C, s2C and s2D.

5) I suggest authors to include the simple schematic of the mechanism they are proposing in the manuscript.

   We thank the reviewer for this helpful suggestion. To improve clarity, in the revised manuscript, we have now included a schematic of the mechanism suggested by our findings. Please refer to Figure s5B in the revised manuscript.

Reference

Bhosle, V.K., Mukherjee, T., Huang, Y.W., Patel, S., Pang, B.W.F., Liu, G.Y., Glogauer, M., Wu, J.Y., Philpott, D.J., Grinstein, S., *et al.* (2020). SLIT2/ROBO1-signaling inhibits macropinocytosis by opposing cortical cytoskeletal remodeling. Nat Commun *11*, 4112.
Blockus, H., and Chédotal, A. (2016). Slit-Robo signaling. Development *143*, 3037-3044.
Coleman, H.A., Labrador, J.P., Chance, R.K., and Bashaw, G.J. (2010). The Adam family metalloprotease Kuzbanian regulates the cleavage of the roundabout receptor to control axon repulsion at the midline. Development (Cambridge, England) *137*, 2417-2426.
Mukovozov, I., Huang, Y.W., Zhang, Q., Liu, G.Y., Siu, A., Sokolskyy, Y., Patel, S., Hyduk, S.J., Kutryk, M.J., Cybulsky, M.I., *et al.* (2015). The Neurorepellent Slit2 Inhibits Postadhesion Stabilization of Monocytes Tethered to Vascular Endothelial Cells. J Immunol *195*, 3334-3344.
Patel, S., Huang, Y.W., Reheman, A., Pluthero, F.G., Chaturvedi, S., Mukovozov, I.M., Tole, S., Liu, G.Y., Li, L., Durocher, Y., *et al.* (2012). The cell motility modulator Slit2 is a potent inhibitor of platelet function. Circulation *126*, 1385-1395.
Tole, S., Mukovozov, I.M., Huang, Y.W., Magalhaes, M.A., Yan, M., Crow, M.R., Liu, G.Y., Sun, C.X., Durocher, Y., Glogauer, M., *et al.* (2009). The axonal repellent, Slit2, inhibits directional migration of circulating neutrophils. Journal of leukocyte biology *86*, 1403-1415.

August 10, 2026

RE: Life Science Alliance Manuscript #LSA-2024-03190R

Dr. Lisa A. Robinson
Hospital for Sick Children
Program in Cell Biology
555 University Avenue, Room 5265 The Hospital for Sick Children
Toronto, Ontario M5G 1X8
Canada

Dear Dr. Robinson,

Thank you for submitting your revised manuscript entitled "APC regulates NSLIT2-induced internalization and signaling of the chemorepellent receptor, ROBO1". We returned this work to two of the original reviewers whose comments are below. As you will see, these reviewers are overall supportive of the changes in place and recommend publication. We appreciate the detailed comments of Reviewer 2 and we agree their concerns on consistency and accuracy in describing your results must be resolved. Regarding their request on replicates and statistics for the PLA data, we invite you to use SuperPlots to display these data (see https://doi.org/10.1083/jcb.202001064 and https://doi.org/10.1091/mbc.E20-09-0583). With these minor revisions and the formatting changes found below, we would be happy to publish your paper in Life Science Alliance.

When submitting your revised manuscript, please include a cover letter addressing the points of Reviewer 2. Please also provide a rebuttal letter responding to the original comments of Reviewer 3.

MANUSCRIPT ORGANIZATION AND FORMATTING:

To avoid unnecessary delays in the acceptance and publication of your paper, please read the following information carefully. Full guidelines are available on our Instructions for Authors page, https://www.life-science-alliance.org/authors

Submission of a paper that does not conform to Life Science Alliance guidelines will delay the acceptance of your manuscript.

-Please upload all your Tables in editable .doc or Excel format.
-Please add the X and Bluesky handles of your host institute/organization, as well as your own, and/or one of the authors, in our system.
-Please be sure that the authorship listing and order are correct and match between the system and the manuscript file.
-Please move the Authors' Contributions section and the Conflict of Interest statement below the Data Availability section in the manuscript file. Additionally, please rename the heading "Competing Interests" to "Conflict of Interest."
-Please consult our manuscript preparation guidelines https://www.life-science-alliance.org/manuscript-prep and make sure your manuscript sections are in the correct orde.
-Please move your main, supplementary figure, and table legends in the main manuscript text after the references section.
-A "Data Availability" section should be placed after the Materials & Methods section. We suggest you state the accession information for the mass spec results in this section. Please consult our guidelines at https://www.life-science-alliance.org/manuscript-prep#format
-Please include an ethics statement indicating approval for the use of human subjects for PBMC isolation.
-Please add a callout for Figure S2D to your main manuscript text.
-Please add molecular weight markers to the blots in Figure 2C.

We encourage our authors to provide original source data, particularly uncropped/-processed electrophoretic blots and spreadsheets for the main figures of the manuscript. If you would like to add source data, we would welcome one PDF/Excel-file per figure for this information. These files will be linked as supplementary "Source Data" files.

It is Life Science Alliance policy that if requested, original data images must be made available to the editors. Failure to provide original images upon request will result in unavoidable delays in publication. Please ensure that you have access to all original data images prior to final submission.

If you are planning a press release on your work, please inform us immediately to allow informing our production team and scheduling a release date.

We welcome submissions of potential cover images for the issue of LSA in which your work would appear. If you have high quality images associated with this work, please feel free to email these, with a caption, to the journal office.

LSA encourages authors to provide a 30-60 second video where the study is briefly explained. These videos will be appear embedded with the manuscript online at Life Science Alliance, and on social media to promote the published paper and authors (for examples, see https://docs.google.com/document/d/1-UWCfbE4pGcDdcgzcmiuJl2XMBJnxKYeqRvLLrLSo8s/edit?usp=sharing). Corresponding or first-authors are welcome to submit the video. Please submit only one video per manuscript. The video can be emailed to contact@life-science-alliance.org

FINAL FILES:

To upload the final version of your manuscript, please log in to your account: https://lsa.msubmit.net/cgi-bin/main.plex
You will be guided to complete the submission of your revised manuscript and to fill in all necessary information. Please get in touch in case you do not know or remember your login name.

The following items are required for acceptance.

-- An editable version of the final text (.DOC or .DOCX) is needed for copyediting (no PDFs).

-- High-resolution figure, supplementary figure and video files uploaded as individual files: See our detailed guidelines for preparing your production-ready images, https://www.life-science-alliance.org/authors

-- Summary blurb (enter in submission system): A short text summarizing in a single sentence the study (max. 200 characters including spaces). This text is used in conjunction with the titles of papers, hence should be informative and complementary to the title. It should describe the context and significance of the findings for a general readership; it should be written in the present tense and refer to the work in the third person. Author names should not be mentioned.

The license to publish form must be signed before your manuscript can be sent to production. A link to the license to publish form will be available to the corresponding author only. Please take a moment to check your funder requirements.

Reviews, decision letters, and point-by-point responses associated with peer-review at Life Science Alliance will be published, alongside the manuscript. If you do want to opt out of having the reviewer reports and your point-by-point responses displayed, please let us know immediately.

Thank you for your attention to these final processing requirements. Please revise and format the manuscript and upload materials as soon as you are able.

Thank you for this interesting contribution to the literature. We look forward to publishing your paper in Life Science Alliance.

Sincerely,

Tim Fessenden
Scientific Editor
Life Science Alliance

Reviewer #1 (Comments to the Authors (Required)):

I have carefully reviewed the revised manuscript and the authors' responses to my comments. I appreciate the authors' efforts to address the concerns raised during the initial review and to provide additional experimental data and clarifications where appropriate. The authors have satisfactorily addressed the major points raised in my previous report, including the additional PLA experiments and clarification of the technical limitations associated with assessing ROBO1-APC association. The authors have appropriately acknowledged the limitations of the PLA approach and have refrained from making conclusions that cannot be directly supported by the available data. The additional experiments and revisions have improved the clarity and overall strength of the manuscript. I find that the authors have adequately addressed my comments and have incorporated the relevant clarifications into the revised manuscript. I therefore have no further major concerns regarding the points raised in my previous review.

Reviewer #2 (Comments to the Authors (Required)):

The main point of the paper is the BioID-based identification of APC as negative regulator of ROBO1 endocytosis which dissociates upon SLIT2 binding, thereby allowing for endocytosis. Unfortunately, the authors were not able to confirm the interaction between APC and ROBO1 via biochemical assays. However, also in absence of biochemical evidence the authors´

finding of the regulatory role of APC in regards to ROBO1 endocytosis is of interest to the readership of Life Science Alliance. Moreover, the authors have performed a number of additional control experiments to strengthen their data, have added to the methods and discussion section to make it easier for the reader to follow the experiments and the conclusions that can be drawn. Finally, they have also addressed all my previous minor comments to make the manuscript more consistent.
However, reading the manuscript again after this long time, I came across some points that should still be addressed (see below). Therefore, I recommend publication in Life Science Alliance pending some minor revisions to text, statistics and figures:

Comments to figures and statistics:

- Figure 2D: Using 3 colours in addition to shape would make it easier to distinguish the three KD conditions.
- Figure 3E-G: I am confused by the labeling underneath the columns where it says "transfected cells" to the left and then "+" oder "-" underneath the bars. From the legend or the representative images it is not apparent that there were any non transfected cells in the experiment. Please clarify in the legend.
- Figure 4 and s4: Like for the previous experiments, the statistics need to be done on the N=3 independent experiments to be really meaningful rather than on individual cells or images. It should also be clearly stated what the individual dots in the graphs represent. While it is nice to also depict the results for individual cells, the averages per experiment need to be shown as well and need to be the ones on which the statistics are based.
- Figure 4E: The figure would be easier to grasp if the antibody combination were additionaly added on top as in the other figure panels.
- Figure 5B: Here, the authors show only statistics for comparing the scrambled conditions with each other, while the main point should be comparing the scrambled condition to the siAPC KD condition. In regards to this, the authors only note "endocytosis was similar in the presence and absence of NSLIT2". This makes sense since NSLIT2 is supposed to dissociate APC, and if there is no APC to start with, this should give the same result. However, is not the most important point that in the vehicle and CSLIT2 condition endocytosis should be higher in the siAPC case than in the siScr case since APC is dissociated due to its depletion mimicking what NSLIT2 would do?? It looks to me like there is a clear trend in this direction supporting this assumption, but maybe too much variability in the data to see a statistical significance (which might be remedied by a higher N...). Still, the authors should report the result of the statistical comparisons between siScr and siAPC and discuss the expectation and outcome clearly in the results section in order to not confuse the reader by omitting this discussion.
- Later in the discussion the authors write (p. 20 line 547): "We further noted that knockdown of APC resulted in more constitutive endocytosis compared to that seen in cells exposed to scrambled siRNA". I assume that this refers to figure 5B where it is (so far) NOT shown with a statistically significant result that siAPC leads to more internalized ROBO! Please amend the statistical testing or your statement along the lines discussed above.
- Figure 6C: The brackets underneath the graph should more clearly comprise also the 2nd vehicle condition.
- Figure 6C: Maybe I am confused: My understanding was that NSlit works by dissociating APC from ROBO, thereby promoting ROBO endocytosis and thus inhibiting cell spreading. So, if APC is not there to start with (siAPC), there should already be an inhibition of cell spreading, and adding NSlit should have the same effect as depleting APC. Instead, in the depicted experiment, depleting APC keeps NSlit from inhibiting spreading. That does not make sense to me in a straightforward manner, but might of course be due to compensatory effects. If I am not mistaken in my reasoning, the assumptions vs results should be better explained to the reader.
- Scheme: I am very much in favor of including a scheme, but would suggest some improvements: ROBO1 starts off being bound to AP2 and clathrin as well as APC. The arrow then points to an NSLIT2 bound ROBO1 from which APC is dissociating, but also AP2 and clathrin are lost which does not make any sense since they are needed and back again in the next piece of the cartoon. So 1) keep AP2 and clathrin associated in the scheme where APC leaves. 2) I think it would further clarify things to show with a dashed inhibitory arrow that ROBO-associated APC somehow inhibits the endocytic components. 3) It would be helpful to add a dashed arrow going out from the clathrin-coated pit to indicate that ROBO1 endocytosis promotes the inhibition of cell spreading.

Minor text edits:

- p14 line 362: There should be an "s" before "2B" to distinguish it from main figure 2B.
- p14 line 368: There should be an "s" before "2D" to distinguish it from main figure 2D.

Response to Reviewers

Manuscript number: LSA-2024-03190
Corresponding author: Lisa A. Robinson

We sincerely thank the reviewers for their comprehensive and constructive feedback. Below, we submit our point-by-point response (in bold) to the reviewers' comments and revisions addressed in this submission.

Reviewer #1

I have carefully reviewed the revised manuscript and the authors' responses to my comments. I appreciate the authors' efforts to address the concerns raised during the initial review and to provide additional experimental data and clarifications where appropriate. The authors have satisfactorily addressed the major points raised in my previous report, including the additional PLA experiments and clarification of the technical limitations associated with assessing ROBO1-APC association. The authors have appropriately acknowledged the limitations of the PLA approach and have refrained from making conclusions that cannot be directly supported by the available data. The additional experiments and revisions have improved the clarity and overall strength of the manuscript. I find that the authors have adequately addressed my comments and have incorporated the relevant clarifications into the revised manuscript. I therefore have no further major concerns regarding the points raised in my previous review.

We thank the reviewer for their careful evaluation of the revised manuscript and constructive feedback throughout the review process. We greatly appreciate their positive assessment of our revisions.

Reviewer #2

The main point of the paper is the BioID-based identification of APC as negative regulator of ROBO1 endocytosis which dissociates upon SLIT2 binding, thereby allowing for endocytosis. Unfortunately, the authors were not able to confirm the interaction between APC and ROBO1 via biochemical assays. However, also in absence of biochemical evidence the authors´ finding of the regulatory role of APC in regards to ROBO1 endocytosis is of interest to the readership of Life Science Alliance. Moreover, the authors have performed a number of additional control experiments to strengthen their data, have added to the methods and discussion section to make it easier for the reader to follow the experiments and the conclusions that can be drawn. Finally, they have also addressed all my previous minor comments to make the manuscript more consistent.
However, reading the manuscript again after this long time, I came across some points that should still be addressed (see below). Therefore, I recommend publication in Life Science Alliance pending some minor revisions to text, statistics and figures:

We thank Reviewer 2 for the careful re-evaluation of our manuscript and for the constructive comments. We appreciate the reviewer's recognition that the additional experiments and clarifications have strengthened the manuscript. We have addressed each of the remaining points as detailed below.

Comments to figures and statistics:

• Figure 2D: Using 3 colours in addition to shape would make it easier to distinguish the three KD conditions.

We thank the reviewer for this helpful suggestion. We have revised Figure 2D to distinguish the three knockdown conditions using different colours and shapes.

• Figure 3E-G: I am confused by the labeling underneath the columns where it says "transfected cells" to the left and then "+" oder "-" underneath the bars. From the legend or the representative images it is not apparent that there were any non transfected cells in the experiment. Please clarify in the legend.

We thank the reviewer for identifying this ambiguity. We have now revised the figure legend 3F and 3G to explicitly define the "+" and "−" conditions. The text of the legend to Figure 3E, 3F and 3G have been revised to describe the experimental conditions more explicitly.

• Figure 4 and s4: Like for the previous experiments, the statistics need to be done on the N=3 independent experiments to be really meaningful rather than on individual cells or images. It should also be clearly stated what the individual dots in the graphs represent. While it is nice to also depict the results for individual cells, the averages per experiment need to be shown as well and need to be the ones on which the statistics are based.

We thank the reviewer for this important point. The statistical analyses and graphical presentation of Figures 4B, 4D, 4E, s4B, s4D and s4E have been revised, and the corresponding figure legends have been clarified.

We have revised the analysis of Figures 4 and s4 so that statistical comparisons are performed using the independent experiments (N = 3 or N=4), with each experiment contributing one value per experimental condition. Thus, the statistical analyses are based on the average measurement calculated for each experiment, rather than on individual cells or images.

The individual-cell measurements are retained in the graphical presentation of the Superplots to illustrate the distribution of the data, while the larger symbols represent the mean of each independent experiment.

• Figure 4E: The figure would be easier to grasp if the antibody combination were additionaly added on top as in the other figure panels.

We thank the reviewer for this helpful suggestion. Figure 4E has been revised; and the antibody-combination labels are added to the top of Figure 4E

• Figure 5B: Here, the authors show only statistics for comparing the scrambled conditions with each other, while the main point should be comparing the scrambled condition to the siAPC KD condition. In regards to this, the authors only note "endocytosis was similar in the presence and absence of

NSLIT2". This makes sense since NSLIT2 is supposed to dissociate APC, and if there is no APC to start with, this should give the same result. However, is not the most important point that in the vehicle and CSLIT2 condition endocytosis should be higher in the siAPC case than in the siScr case since APC is dissociated due to its depletion mimicking what NSLIT2 would do?? It looks to me like there is a clear trend in this direction supporting this assumption, but maybe too much variability in the data to see a statistical significance (which might be remedied by a higher N...). Still, the authors should report the result of the statistical comparisons between siScr and siAPC and discuss the expectation and outcome clearly in the results section in order to not confuse the reader by omitting this discussion.

We thank the reviewer for highlighting this important point. We agree that the comparison between siScr and siAPC is key to the interpretation of this experiment because APC depletion is expected to mimic, at least in part, the effect of SLIT2-induced APC dissociation.

We have added the requested statistical comparisons between the siScr and siAPC conditions for the relevant experimental conditions in Figure 5B. Importantly, APC depletion significantly increased ROBO1 internalization (p=0.0078 siScr vs siAPC).

We have also revised the Results section to explain more clearly the expected relationship between APC depletion and SLIT2 treatment. In particular, we now state that APC depletion is expected to relieve the inhibitory constraint imposed by APC on ROBO1 endocytosis and thereby increase ROBO1 internalization. Thus, APC depletion may, at least in part, phenocopy the effect of SLIT2-induced APC dissociation. We have included this in the Results section of main manuscript, page 8 lines 216 -220.

• Later in the discussion the authors write (p. 20 line 547): "We further noted that knockdown of APC resulted in more constitutive endocytosis compared to that seen in cells exposed to scrambled siRNA". I assume that this refers to figure 5B where it is (so far) NOT shown with a statistically significant result that siAPC leads to more internalized ROBO! Please amend the statistical testing or your statement along the lines discussed above.

We thank the reviewer for pointing this out. The statement did refer to the experiment shown in Figure 5B. As noted in our response above, we have now added the statistical comparison between the siScr and siAPC conditions. This analysis showed that APC knockdown significantly increased ROBO1 internalization compared with scrambled siRNA (p = 0.0078, siScr vs. siAPC). We have therefore retained the statement in the Discussion and revised the corresponding text in the Results section to report this statistical comparison and clarify that the observed increase in ROBO1 internalization is statistically significant.

• Figure 6C: The brackets underneath the graph should more clearly comprise also the 2nd vehicle condition.

We thank the reviewer for pointing this out. We have revised Figure 6C accordingly.

• Figure 6C: Maybe I am confused: My understanding was that NSlit works by dissociating APC from ROBO, thereby promoting ROBO endocytosis and thus inhibiting cell spreading. So, if APC is not there to start with (siAPC), there should already be an inhibition of cell spreading, and adding NSlit should have the same effect as depleting APC. Instead, in the depicted experiment, depleting APC keeps NSlit from inhibiting spreading. That does not make sense to me in a straightforward manner, but might of course be due to compensatory effects. If I am not mistaken in my reasoning, the assumptions vs results should be better explained to the reader.

We thank the reviewer for highlighting this important point. Our results show that, although both APC depletion and NSLIT2 treatment affect ROBO1 endocytosis, they do not produce the same effect on cell spreading.

As shown in Figure 5B, APC depletion significantly increases constitutive ROBO1 internalization ($p = 0.0078$, siScr vs. siAPC). However, as shown in Figure 6C, APC depletion does not reproduce the inhibitory effect of NSLIT2 on cell spreading. Moreover, in APC-depleted cells, NSLIT2 is no longer able to inhibit cell spreading. Thus, increased constitutive ROBO1 endocytosis following APC depletion is not, by itself, sufficient to inhibit cell spreading, and APC is nevertheless required for the inhibitory effect of NSLIT2.

We have revised the Results section to clarify this important point. We now explain that the effect of NSLIT2 on cell spreading may depend not simply on the overall amount of ROBO1 internalization, but on the regulated internalization of a specific pool of ROBO1 or on the spatial and/or temporal regulation of ROBO1 endocytosis. Such regulated endocytosis may be necessary to engage the downstream signaling or cytoskeletal changes that inhibit cell spreading. Please refer to Results section of main manuscript page 9 lines 234-242

• Scheme: I am very much in favor of including a scheme, but would suggest some improvements: ROBO1 starts off being bound to AP2 and clathrin as well as APC. The arrow then points to an NSLIT2 bound ROBO1 from which APC is dissociating, but also AP2 and clathrin are lost which does not make any sense since they are needed and back again in the next piece of the cartoon. So 1) keep AP2 and clathrin associated in the scheme where APC leaves. 2) I think it would further clarify things to show with a dashed inhibitory arrow that ROBO-associated APC somehow inhibits the endocytic components. 3) It would be helpful to add a dashed arrow going out from the clathrin-coated pit to indicate that ROBO1 endocytosis promotes the inhibition of cell spreading.

We thank the reviewer for these very helpful suggestions. We have revised the model scheme in Figure s5B to improve its consistency with the proposed mechanism. Specifically:

i. **AP2 and clathrin are now retained in association with ROBO1 during the step in which APC dissociates following NSLIT2 stimulation.**
ii. **A dashed inhibitory arrow has been added to indicate the proposed inhibitory effect of ROBO-associated APC on the endocytic machinery.**
iii. **A dashed arrow has been added from the clathrin-coated pit toward inhibition of cell spreading, illustrating the proposed functional consequence of ROBO1 internalization.**

Minor text edits:
• p14 line 362: There should be an "s" before "2B" to distinguish it from main figure 2B.
• p14 line 368: There should be an "s" before "2D" to distinguish it from main figure 2D.

We apologize for the oversight. In the revised manuscript, we have corrected these typos. Please see page 4 line 98 and line 104.

October 16, 2026

RE: Life Science Alliance Manuscript #LSA-2024-03190RR

Dr. Lisa A. Robinson
Hospital for Sick Children
Program in Cell Biology
555 University Avenue, Room 5265 The Hospital for Sick Children
Toronto, Ontario M5G 1X8
Canada

Dear Dr. Robinson,

Thank you for submitting your Research Article entitled "APC regulates NSLIT2-induced internalization and signaling of the chemorepellent receptor, ROBO1". It is a pleasure to let you know that your manuscript is now accepted for publication in Life Science Alliance. Congratulations on this interesting work.

The final published version of your manuscript will be deposited by us to PubMed Central upon publication.

Your manuscript will now progress through copyediting and proofing. It is journal policy that authors provide original data upon request.

Reviews, decision letters, and point-by-point responses associated with peer-review at Life Science Alliance will be published, alongside the manuscript. If you do want to opt out of having the reviewer reports and your point-by-point responses displayed, please let us know immediately.

IMPORTANT: If you will be unreachable at any time, please provide us with the email address of an alternate author. Failure to respond to routine queries may lead to unavoidable delays in publication.

Scheduling details will be available from our production department. You will receive proofs shortly before the publication date. Only essential corrections can be made at the proof stage so if there are any minor final changes you wish to make to the manuscript, please let the journal office know now.

DISTRIBUTION OF MATERIALS:
Authors are required to distribute freely any materials used in experiments published in Life Science Alliance. Authors are encouraged to deposit materials used in their studies to the appropriate repositories for distribution to researchers.

You can contact the journal office with any questions, contact@life-science-alliance.org

Again, congratulations on a very nice paper. I hope you found the review process to be constructive and are pleased with how the manuscript was handled editorially. We look forward to future exciting submissions from your lab.

Sincerely,

Tim Fessenden
Scientific Editor
Life Science Alliance
