# Supplementary material for: APC regulates NSLIT2-induced internalization and signaling of the chemorepellent receptor, ROBO1
Source: Life Sci Alliance. 2026 Nov 10;10(1):e202403190. doi: 10.26508/lsa.202403190 (PMC13755696; doi:10.26508/lsa.202403190)
Supplement: Supplementary file 2 [file LSA-2024-03190_TableS1.docx]

**Table s1. Antibodies**

| **Antibody** | **Company and Catalogue Number** | **RRID** | **Working Condition** |
| --- | --- | --- | --- |
| Anti-ROBO1 | Thermo Fisher Scientific  PA5-29917 | AB_2547391 | WB: 2.52 µg/mL  4°C, overnight |
| Anti-ROBO1 | Sigma-Aldrich  HPA052968 | AB_2682001 | PLA: 1 µg/mL  4°C, overnight |
| Anti-ROBO1 | R&D Systems  MAB7118 | AB_3658702 | PLA: 10 µg/mL  4°C, overnight |
| Anti-APC (CC-1) | Abcam  ab16794 | AB_443473 | PLA: 10 µg/mL  4°C, overnight |
| Anti-APC (EP701Y) | Abcam  ab40778 | AB_2057497 | PLA: 25 µg/mL  4°C, overnight |
| Anti-clathrin (D3C6) | Cell Signaling Technology 4796 | AB_10828486 | WB: 0.0665 µg/mL  4°C, overnight |
| Anti-clathrin (X22) | Abcam  ab2731 | AB_303256 | PLA: 30 µg/mL  4°C, overnight |
| Anti-β-actin | ABclonal  AC004 | AB_2737399 | WB: 0.8785 µg/mL  4°C, overnight |
| Anti-FLAG (M2) | Sigma-Aldrich  F1804 | AB_262044 | IF: 1.8 µg/mL  room temperature, 1 h |
| Rabbit Anti-FLAG | Cell Signaling  2368S | AB_2217020 | PLA: 0.168 µg/mL  4°C, overnight |
| Anti-p38 MAPK | Cell Signaling  9212 | AB_330713 | PLA: 0.68 µg/mL  4°C, overnight |
| Peroxidase AffiniPure goat anti-mouse IgG (H+L) | Jackson ImmunoResearch Laboratories  115-035-003 | AB_10015289 | WB: 0.16 µg/mL  room temperature, 1 h |
| Peroxidase AffiniPure goat anti-rabbit IgG (H+L) | Jackson ImmunoResearch Laboratories  111-035-144 | AB_2307391 | WB: 0.16 µg/mL  room temperature, 1 h |
| Alexa fluor 488- AffiniPure Fab fragment goat anti-mouse IgG | Jackson ImmunoResearch Laboratories  115-547-003 | AB_2338869 | IF: 2.13 µg/mL  room temperature, 45 min |
